# High-dimensional single-cell analysis unveils distinct immune signatures of peripheral blood in patients with pancreatic ductal adenocarcinoma

**DOI:** 10.3389/fendo.2023.1181538

**Published:** 2023-06-06

**Authors:** Yu Pan, Jianfeng Gao, Jiajing Lin, Yuan Ma, Zelin Hou, Yali Lin, Shi Wen, Minggui Pan, Fengchun Lu, Heguang Huang

**Affiliations:** ^1^ Department of General Surgery, Fujian Medical University Union Hospital, Fuzhou, China; ^2^ Department of Oncology and Hematology and Division of Research, Kaiser Permanente, Santa Clara, CA, United States

**Keywords:** pancreatic ductal adenocarcinoma, single-cell sequencing, T-cell receptor immune signatures, tumor, signatures calculation

## Abstract

**Introduction:**

Pancreatic ductal adenocarcinoma (PDAC) is one of the most lethal malignancies with poor response to immune checkpoint inhibitors. The mechanism of such poor response is not completely understood.

**Methods:**

We assessed T-cell receptor (TCR) repertoire and RNA expression at the single-cell level using high-dimensional sequencing of peripheral blood immune cells isolated from PDAC patients and from healthy human controls. We validated RNA-sequencing data by performing mass cytometry (CyTOF) and by measuring serum levels of multiple immune checkpoint proteins.

**Results:**

We found that proportions of T cells (CD45+CD3+) were decreased in PDAC patients compared to healthy controls, while proportion of myeloid cells was increased. The proportion of cytotoxic CD8+ T cells and the level of cytotoxicity per cell were increased in PDAC patients, with reduced TCR clonal diversity. We also found a significantly enriched S100A9+ monocyte population and an increased level of TIM-3 expression in immune cells of peripheral blood in PDAC patients. In addition, the serum level of soluble TIM-3 (sTIM-3) was significantly higher in PDAC patients compared to the non-PDAC participants and correlated with worse survival in two independent PDAC cohorts. Moreover, sTIM-3 exhibited a valuable role in diagnosis of PDAC, with sensitivity and specificity of about 80% in the training and validation groups, respectively. We further established an integrated model by combining sTIM-3 and carbohydrate antigen 19- 9 (CA19-9), which had an area under the curve of 0.974 and 0.992 in training and validation cohorts, respectively.

**Conclusion:**

Our RNA-seq and proteomic results provide valuable insight for understanding the immune cell composition of peripheral blood of patients with PDAC.

## Introduction

Pancreatic ductal adenocarcinoma (PDAC) is one of the most lethal malignancies with five-year overall survival (OS) of approximately 9% (1). The tumor microenvironment (TME) of PDAC is also uniquely characterized by a dense desmoplastic stroma that is related to treatment resistance and local immune suppression (2, 3). Recent studies have shown that combination immunotherapy may improve the efficacy for PDAC (4, 5). In our previous study, we had found that increased CD47 expression of PDAC cells and increased tumor infiltrating macrophages in the TME correlated with poor survival. We also found that targeting CD47 resulted in increased inflammatory infiltrates in the TME and enhanced response to immune checkpoint inhibitor (6).

Several studies have shown that cancer can cause perturbation of hematopoietic stem cells, increase the proliferation of monocytic and granulocytic cells that move into the TME of tumors, and differentiate into tumor infiltrating macrophages that suppress inflammatory anti-tumor response (7, 8). Depletion of peripheral blood lymphocytes has been shown to be a major immunologic feature of metastatic pancreatic cancer. Blood immunologic biomarkers such as immunosuppressive cytokines IL-6 and IL-10, or cellular biomarkers CTLA-4 and TIM3 have been associated with OS and progression in patients with PDAC (9). Serum levels of soluble PD-1 and PD-L1 (sPD-1/sPD-L1), released from the surface of PD-1/PD-L1-expressing cells that may reflect PD-1/PD-L1 levels, can be detected in the serums of cancer patients with prognostic values (10, 11). Kruger et al. reported that sPD-1 and sPD-L1 are markers of systemic inflammation in patients with unresectable pancreatic cancer, but they were not associated with the OS (12).

T cell-mediated antigen recognition depends on the interaction of T cell receptor (TCR) with antigen major histocompatibility complex (MHC) molecules (13, 14). TCR is highly diverse heterodimers consisting of α and β chains (αβ TCR) expressed by most T cells. The variable regions of TCR α and δ chains are encoded by multiple variable (V) and linked (J) genes, while TCR β and γ chains are encoded by diversity (D) genes (15, 16). The V region of TCR (Vα and Vβ) has three hypervariable regions, complementary determination region 1 (CDR1), CDR2 and CDR3, among which CDR3 has the largest variation, which directly determines the specificity and diversity of TCR (17). Therefore, high-throughput sequencing of TCR CDR3 may reflect the adaptive immune status and is helpful to understand the mechanism of anti-tumor immunity.

A previous study using bulk TCR sequencing of peripheral blood revealed different immune responses between pancreatic cancer and benign pancreatic diseases (18), however, the relationship between immune cell composition and clonal diversity of TCR was not investigated. Identifying the diversity of TCR to each T cell may help us to better understand the mechanism of tumor immune response. We performed high-dimensional single-cell sequencing of peripheral blood mononuclear cells (PBMCs) from PDAC patients and normal controls using 10× Genomics RNA/TCR sequencing and mass cytometry technology, with unsupervised clustering of immune cells and defining cell subclusters, revealing the proportion and genetic characteristics of cell subclusters, and recognizing differential gene and pathways. We explored the characteristics of peripheral blood adaptive immune response at single-cell level. In addition, we measured multiple serum immune-related soluble proteins to gain insight into disease-related immune responses and provide potential valuable targets for immune targeting.

## Materials and methods

### Patients

Peripheral blood was obtained from patients at Fujian Medical University Union Hospital, Fuzhou, China, from June 2018 to December 2021. Pancreatic cancer (PC) patient inclusion criteria: (1) patients had histologically confirmed PDAC; (2) aged 18 years or older. Exclusion criteria: (1) patients with neoadjuvant treatment; (2) with inflammatory diseases; (3) with active infection. Blood samples were collected prior to surgery. All patients were followed up for survival status until September 2022. The stages of patients were assessed based on the American Joint Committee on Cancer version 8 (AJCC 8). Informed consent was signed before sample collection. Pancreatic benign diseases included chronic pancreatitis, neuroendocrine tumors, and cystic neoplasms. Blood samples were collected from healthy individuals during routine physical examination. The health status of the volunteers is determined by reviewing physical examination results and medical history. Exclusion criteria included: acute infection; cancer-related history; organ dysfunction; coagulation dysfunction; immune deficiency syndrome; receiving immunotherapy; blood transfusion records within the past 6 months; use of drugs affecting peripheral blood cells, such as recombinant human erythropoietin and granulocyte stimulating factor, within the past 3 months.

The first cohort for mass cytometry and single-cell sequencing analysis, including 10 PDAC patients and 10 healthy individuals (shown in **Tables S1**, **S2**). The second cohort for for the validation experiments of single-cell sequencing data, including 50 PDAC patients and 50 healthy individuals (shown in **Table S5**). The third cohort for the analysis of the expression of serum soluble immune proteins, including training cohort and validation cohort. The training cohort contained 45 participants with PDAC, 20 participants with benign pancreatic diseases and 30 healthy volunteers (shown in **Table S6**). The validation cohort contained 53 participants with PDAC, 22 participants with benign pancreatic diseases and 25 healthy volunteers (shown in **Table S8**). The study was approved by the Committee for the Ethical Review of Research, Fujian Medical University Union Hospital.

### Blood samples

The peripheral blood mononuclear cells (PBMCs) were isolated from whole blood samples by Ficoll-Hypaque gradient centrifugation (Haoyang Biotech, Tianjin, China) for the single-cell sequencing and mass cytometry analyses. Blood samples were centrifuged at 3000 g for 10 min at 4°C to separate the serum. Serum samples were stored at - 80°C until experiments were performed. Complete blood count (Neutrophils, lymphocytes and monocytes), and carbohydrate antigen 19–9 (CA19-9) were measured at the central laboratory of Fujian Medical University Union Hospital. In this study, scRNA-seq data of sample Con1 and Con2 (19) (doi: 10.3389/fimmu.2021.645666) were obtained from a publicly available cDNA microarray dataset on PBMCs (2 normal peripheral blood, GSE181279), and scRNA-seq data of sample Con3, Con4 and Con5 were obtained from a previous study (20) (https://doi.org/10.1038/s43587-022-00198-9) by Oscar Junhong Luo. (3 normal peripheral blood, GSE157007).

### Single-cell RNA sequencing

The single cell suspensions were converted into the Chromium single cell controller to generate single-cell gel beads in the emulsion, using the single-cell 5′ Library and Gel Bead Kit and Chromium Single Cell A Chip Kit (10× Genomics). A total of approximately 10000 cells to 12000 cells/chip were captured on the 10× Chromium platform. The libraries were pair-end sequenced on Illumina Nobaseq6000 platform with read lengths of 150bp (performed by CapitalBio, Beijing). And all the procedures including the complementary DNA synthesis and library preparation were performed according to the standard manufacturer’s protocol using version 2 chemistry.

### Cell Ranger pipeline

Cell Ranger software version 3.0.1, available from 10x Genomics, were used to process raw sequencing data obtained from the Illumina sequencing output with default and recommended parameters. In short, raw base call (BCL) files were converted to FASTQ files for each sample by CellRanger mkfastq. The FASTQ files were mapped to the GRCh38 human reference genome to distinguish human cell using CellRanger count. Then, feature-barcode matrices were generated for each sample by filtering, barcode counting, and unique molecular identifiers (UMI) counting. The CellRanger aggr pipeline was used to combine the data from five samples into an experiment-wide feature-barcode matrix. Finally, the feature-barcode matrix was loaded to the R package Seurat for quality control and downstream analyses.

### Seurat pipeline

The combined data set were read into the Seurat R package (version 4.0.2). Low quality cells were filtered out according to these thresholds (**Table 1**).

**Table 1:** Single-cell RNA sequencing data quality control standards. (6)

Index	threshold
nUMI	>1000
nUMI	<30000
nGene	>750
nGene	< 3000
Mito.percent	< 10%

Using these thresholds, the number of cells vary as follows (**Table 2**):

**Table 2:** Overview of single-cell RNA sequencing data quality. (6)

	Group	Cell count (Before QC)	Cell count (After QC)	Group cell count	Total cell count
PC1	Pancreatic cancer	8768	6274	26,548	57175
PC2		5562	3609		
PC3		8396	4724		
PC4		5112	4521		
PC5		9885	7420		
Con1	Healthy control	7115	6722	30,627	
Con2		6945	6412		
Con3		6320	5521		
Con4		7069	6333		
Con5		6984	5639		

Then, the data was normalized and scaled through Seurat’s NormalizeData and ScaleData functions. The highly variable gene (HVGs) was identified using the FindVariableGenes function for the next principal component analysis (PCA), with default parameters. PCA was performed based on about 2000 variant genes and the first 14 PCA component were used for the 2D uniform manifold approximation and projection (UMAP) dimension reduction. Cell clusters were identified by running the FindClusters function of Seurat using a resolution of 0.6. The differential test used Wilcoxon ranked-sum method.

### Function analysis for differently expressed genes

DEGs were identified using the FindMarkers function of Seurat. The following cutoff threshold values were used: adj. p val <0.05. In order to better characterize the signature of DEGs, we used a fast per-ranked gene set enrichment analysis (GSEA) named fgsea (v1.11.1, R package) to evaluate functional enrichment analysis for DEGs. The hallmark gene sets from Molecular Signatures Database5 were extracted and used to feed each differentially expressed gene lists.

GSEA analysis was performed to detect which gene set was significantly enriched in each specific cell cluster. Then adjusted P value < 0.05 were selected as the significantly and functionally enriched biological states or processes.

### Single-cell TCR sequencing analysis

Single-cell TCR clonotypes were assembled using Cell Ranger VDJ function. Single-cell barcodes were then used to tie corresponding VDJ (variable-diversity-joining TCR gene segments) and gene expression data simultaneously. Only TCRs with full and paired α and β chain sequences were included in the analysis. TCR Clonotypes were then determined from grouping of cell barcodes that shared the same set of productive CDR3 nucleotide sequences. TCR analysis utilized our previously described scRepertoire R package (v1.805) with clonotype being defined as the combination of the gene components of the VDJ and the nucleotide sequence for both TCRA and TCRB chains and assigned on the integrated Seurat object. The diversities of the TCR CDR3 regions were evaluated by the InvSimpson index and Shannon–Weiner index, respectively. TCR cluster lineage tracing was performed by considering all clonotypes shared by cells from more than one cluster. Raw numbers of cluster clonotype intersections were analyzed and visualized as upset plots.

### Mass cytometry (CyTOF)

A panel of 44 metal isotope-tagged antibodies (**Table S6**) was used to evaluate the immune cell populations in peripheral blood of pancreatic cancer. Fresh PBMCs were washed resuspended, cisplatin cocktail was added and mixed, and incubated for 5min at room temperature under light. 50 μl membrane antibody cocktail was added to each tube, incubated for 1h, then washed, and the supernatant was discarded after centrifugation. 1mL of nuclear antigen staining buffer was added to each tube, and after 30min of incubation, 2mL of nuclear antigen fixing solution was added, and the supernatant was discarded by centrifugation. Each tube was fixed with 1mL formaldehyde working solution, incubated for 10min, and the supernatant was discarded by centrifugation. Cells were resuspended with 10% EQ Four Element Calibration Beads per tube. The samples were detected by Helios 2 CY-TOF mass cytometer. After data collection, Cytometry by Time-of-Flight software (V6.7) was used for merging and data standardization. Data were analyzed in Cytobank (https://www.cytobank.org/) and R package. Normalized marker expression levels were visualized as heatmaps. The cell types were distinguished by canonical markers. The T-SNE map was generated by R package, and the differences in phenotypes and relative proportion of different cell subclusters were analyzed. P < 0.05 was considered statistically significant.

### Serum soluble checkpoint/co-stimulatory biomarkers analysis

A panel of sixteen checkpoint/co-stimulatory biomarkers was analyzed in serum using MILLIPLEX Human Immuno-Oncology Checkpoint Protein Panel Kits (Cat. No. HCKPMAG-11K, Merck Millipore; Massachusetts, US). Samples were performed in duplicate, run in batches. Data were analyzed using the MILLIPLEX Analyst. V5. 1 (Merck Millipore).

### Statistical analysis

Quantitative data were expressed as the mean ± standard deviation (SD) and analyzed based on variance and Student’s t-tests. Chi-square tests were performed to compare serum protein levels and clinical features. Correlative analyses were performed using the Spearman coefficient analysis. Receiver operating characteristics (ROCs) curves were constructed for each biomarker to assess the diagnostic accuracy of the biomarkers in distinguishing pancreatic cancer from normal controls. The area under the ROC curve (AUC) was used to compare the biomarkers for diagnostic purposes. OS was measured from the to the day of death from any cause or the last censored follow-up. Survival was measured using Cox-regression and Kaplan-Meier analysis. Data were analyzed using the Statistical Package for Social Science version 22.0 (SPSS, IBM, Armonk, USA).

## Results

### Single-cell RNA-seq of peripheral blood mononuclear cells of patients with PDAC

To assess the changes of systemic immune state in patients with PDAC, we performed scRNA-seq on PBMCs from five healthy individuals (Con1-Con5) and five treatment-naïve PDAC (PC1-PC5) patients. Sample and patient metadata were shown in **Table S1** and **S2**, and the baseline characteristics of patients and healthy controls were shown in **Table S3**. The PBMCs were subjected to single-cell RNA sequencing (scRNA-seq) using the 10× genomics, data were then processed using Cell-Ranger pipelines. After quality control and filtering, transcriptomic maps of 26,548 and 30,627 CD45 positive cells, of which 10,745 and 21,739 cells had paired TCR clonotypes, were obtained from the PDAC patients and healthy controls, respectively (**Figure 1A**).

**Table 1:**
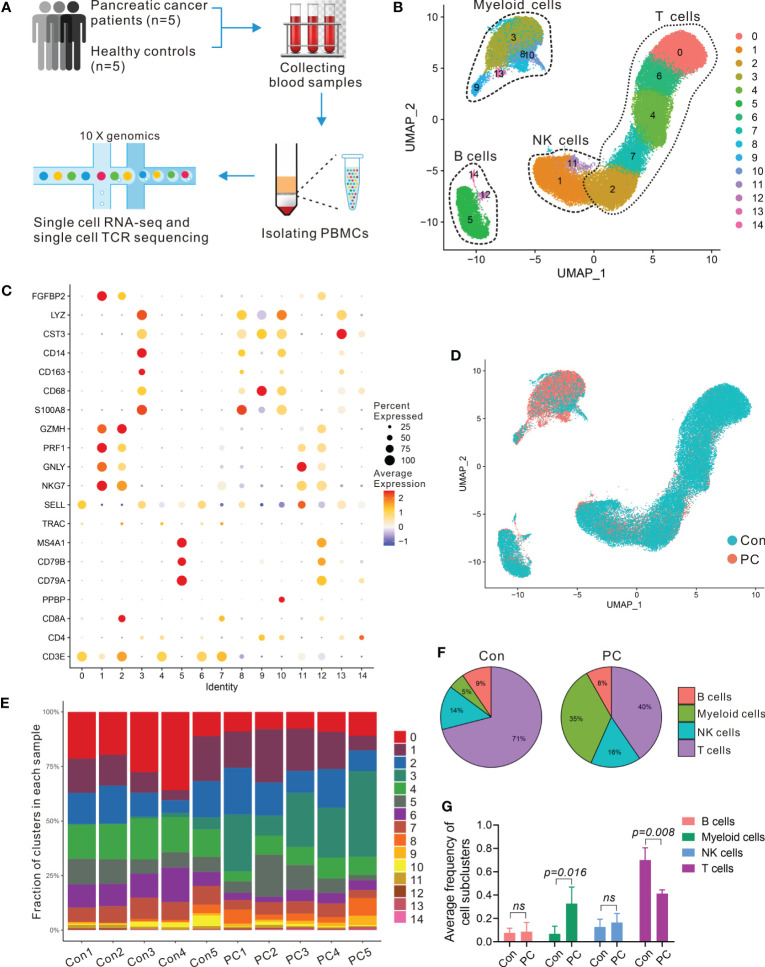
scRNA-seq of PBMCs in PDAC. **(A)** Schematic of single-cell RNA sequencing and TCR-seq of PBMCs from PDAC patients and healthy individuals. **(B)** UMAP plot of all PBMCs from 5 healthy individuals (Con1-Con5) and 5 treatment-naïve PDAC (PC1-PC5). **(C)** Dot plot depicting percent expression and average expression of canonical marker genes in all clusters. **(D)** UMAP plot of PBMCs from PDAC and control group. **(E)** Fraction of 15 immune clusters in each sample from PDAC and control group. **(F, G)** Average proportion of immune subclusters in PDAC and control group. One-way analysis of variance Wilcoxon rank sum test was used.

To enable an unbiased systematic comparison across patients, we merged the data from all healthy individuals and patients to create a map of peripheral blood immune cells by using Seurat standard workflow, then the unsupervised clustering revealing a various set of leukocyte clusters. Comparison with the Cellmarker database and assessment of canonical markers identified the majority of expected immune cell types (**Figure 1B**), including: T cells (cluster 0, 2, 4, 6, and 7) characterized by CD3E and T cell receptor (TRAC) expression; B cells (cluster 5, 12, and14) characterized by *CD79A, CD79B*, and *MS4A1* expression; natural killer (NK) cells (1 and 11 clusters) characterized by *NKG7, FGFBP2*, and *GNLY* expression; five subsets of myeloid cells (cluster 3, 8, 9, 10 and 13) characterized by *CD68, CD14, CST3, LYZ*, and *S100A8* expression, respectively (**Figures 1C**; **S1A**). We further investigated the proportion and composition of immune cell in each group or individual sample by the identified immune cell populations (**Figures 1D, E**; **S1B**). Unlike the healthy controls in which the main immune cells were T cells, the peripheral blood immune cells from PDAC patients had a large proportion of myeloid cells (**Figures 1F, G**; **S1C, D**).

To examine transcriptomic changes in the systemic immune environment of pancreatic cancer, we recognized differentially expressed genes (DEGs) by comparing the patients with pancreatic cancer to the control samples and then performed gene set enrichment analysis (GSEA) of the DEGs in the systemic immune environment. We observed the top 25 upregulated genes in patients compared with controls including *S100A8, S100A9, LYZ, RACK1, VCAN, ATP5MC2, FYB1*, and *FCN1* (**Figure S2A**) and those upregulated genes involved in ATP biosynthetic process, oxidative phosphorylation, proteolysis, innate immune response, inflammatory response, immune cells migration, and apoptosis (**Figures S2B, C**). These results highlighted a strong signature for inflammatory and immune responses in PDAC patients.

**Figure 2:**
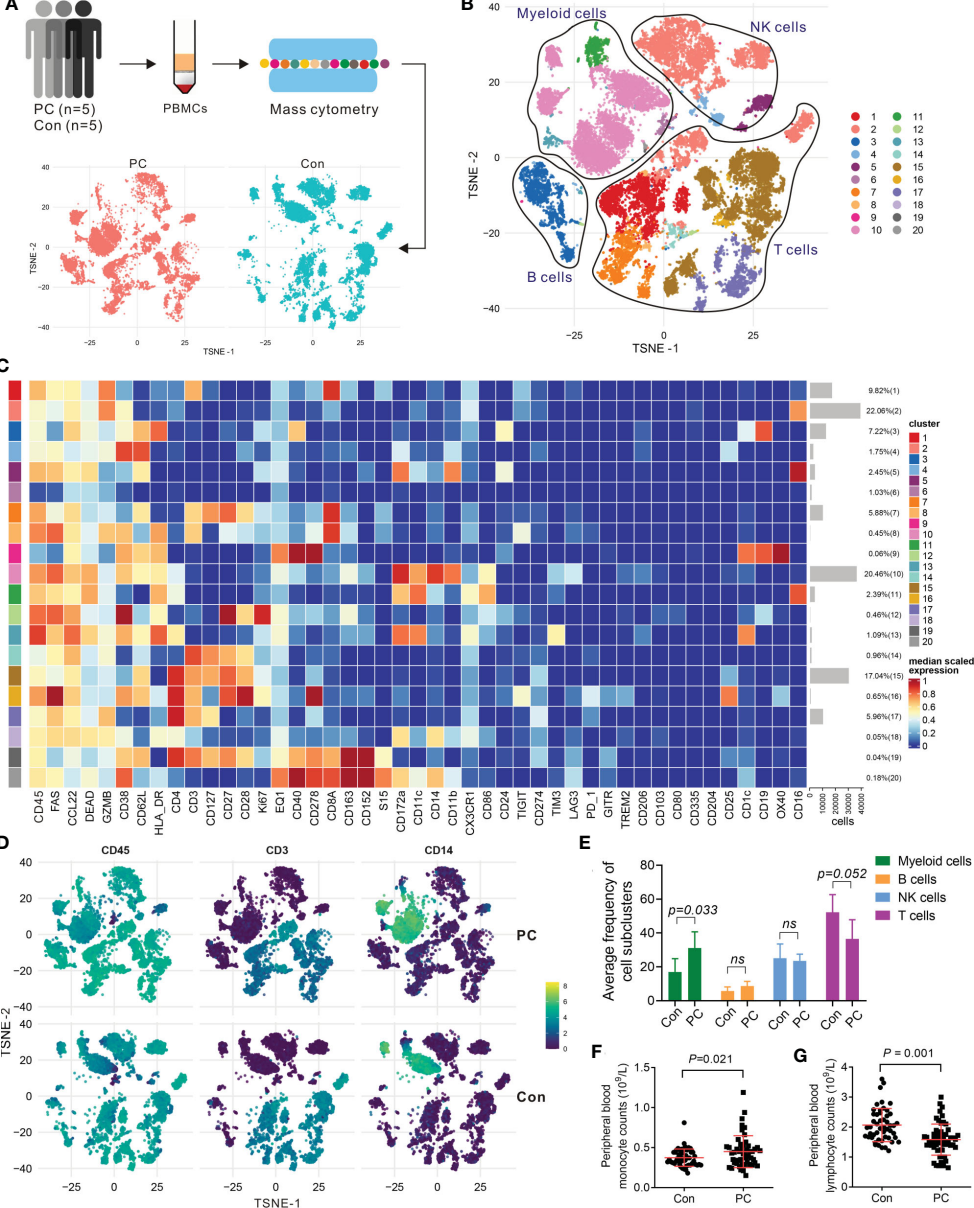
Mass cytometry confirmed the major clusters of PBMCs in PDAC. **(A)** Schematic of workflow for CyTOF (PC, n = 5; Con, n = 5). **(B)** T-SNE plot of immune cell clusters from PC and Con merged. **(C)** Heatmap displaying the relative protein expression of select markers in each cell subcluster. **(D)** T-SNE plot showing the selected markers of CD45, CD3, and CD14 in PC and control group. **(E)** Average proportion of immune subclusters in PC and Con group. One-way analysis of variance Wilcoxon rank sum test was used. **(F)** Cell counts of peripheral blood monocytes in PC and Con group. **(G)** Cell counts of peripheral blood lymphocytes in PC and Con group.

To validate the scRNA-seq data, we confirmed the immune cell surface protein markers in five PDAC patients (PC6-PC10) and five healthy controls (Con6-Con10) by using mass cytometry (CyTOF) (**Figure 2A**), using 44 metal isotope-tagged antibodies (**Table S4**). Patient and control information was shown in **Tables S1**, **S2**. PBMCs were collected for CyTOF analysis from which 20 subpopulations were identified in PDAC patients and controls (**Figure 2B**). The expression of protein markers in each population were shown in **Figure 2C**, and canonical cell types were identified, including CD4^+^ T cells (CD3 and CD4), CD8^+^ T cells (CD3 and CD8), B cells (CD19 and CD24), NK cells (CD16, CD56, and CD335), and myeloid cells (CD14, CD16, CD163, CD11c, CD11b, and HLA-RD) (**Figures 2D**; **S3**). We confirmed that the percentages of these major cell types in PDAC and controls measured by CyTOF were in line with the result of scRNA-seq (**Figures 2E**; **S4A**). To further confirmed the numbers and proportions of lymphocytes and monocytes between PDAC patients and normal healthy controls, we measured the complete blood count from 50 patients with PDAC and 50 healthy volunteers. The baseline characteristics of patients and healthy controls are shown in **Table S5**. We found decreased percent of lymphocytes and increased percent of monocytes in PDAC patients (**Figures 2F, G**; **S4B, C**).

### Expansion of cytotoxic T cells in patients with PDAC

**Figure 3:**
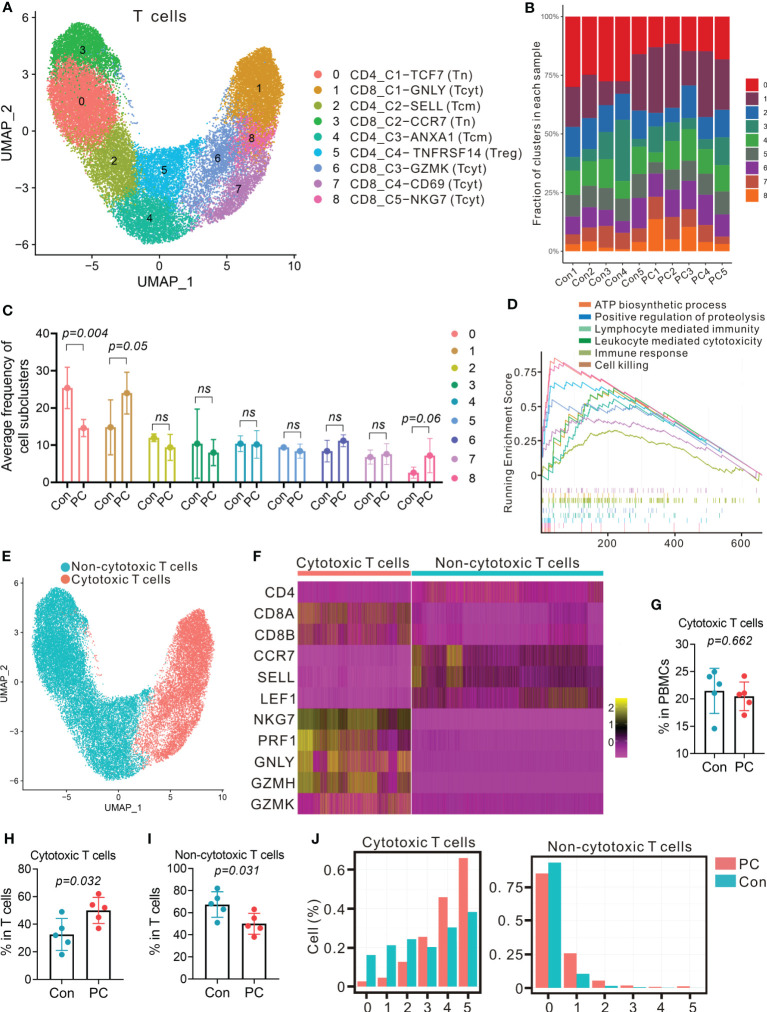
Distinct T-cell transcriptional signatures in PDAC and control group. **(A)** UMAP plot of T cells presenting 9 clusters (Tcm, central memory T cell; Tcyt, cytotoxic T cell; Tn, naïve T cell; Treg, regulatory T cell). **(B)** Fraction of 9 T cell subclusters in each sample from PC patient and control. **(C)** Average proportion of T cell subclusters in PC and control group. One-way analysis of variance Wilcoxon rank sum test was used. **(D)** Gene set enrichment analysis of differentially expressed genes in total T cells from PC versus control group. **(E)** UMAP plot from merged data of T cells. Different colors represent 2 clusters (non-cytotoxic T cells and cytotoxic T cells). **(F)** Heatmap displaying expression of select genes in 2 clusters (non-cytotoxic T cells and cytotoxic T cells). **(G)** Proportion of cytotoxic T cells in total PBMCs from PC and control group. **(H)** Proportion of cytotoxic T cells in T cells from PC and control group. **(I)** Proportion of non-cytotoxic T cells in T cells from PC and control group. **(J)** Number of detected genes out of 5 cytotoxic genes (NKG, PRF1, GNLY, GZMA and GZMB) per cell.

The largest immune cell clusters present in the peripheral blood of healthy individuals and PDAC patients were T cells. To scrutinize and better define the T cell clusters identified by single-cell RNA-seq, we computationally separated T cell clusters (17,019 total cells, **Figure 1B**) and reanalyzed these data. The approach produced 9 distinct T cell clusters broadly defined by the specific marker gene expression (**Figures 3A**; **S5A**), including 5 CD8^+^ and 4 CD4^+^ T cell clusters. We annotate these T-cell subtypes using distinct expression patterns of typical T-cell markers coupled with reference data sets (21–23). CD4_C2-SELL and CD4_C3-ANXA1 correspond to central memory T cells (Tcm) due to the stable expression of distinct signature genes, including *CD69, LEF1, SELL, CCR7*, and *ANXA1*. CD8_C1-GNLY, CD8_C3-GZMK, CD8_C4-CD69, and CD8_C5-NKG7 correspond to cytotoxic CD8 T cells (Tcyt), characterized by expression of *GZMA, GZMB, GZMK, PRF1, GNLY*, and *NKG7*. Moreover, CD4_C1-TCF7 and CD8_C2-CCR7 belong to naïve T cells (Tn) due to the presence of *LEF1, SELL, TCF7*, and *CCR7*, and absence of the effector genes. CD4_C4-TNFRSF14 shows distinct features of regulatory T cell (Treg), such as high expression of *FOXP3, CTLA4, TNFRSF14, TIGIT*, and *ICOS*. All T cell clusters were stably expressed in both PDAC and healthy samples (**Figures 3B**; **S5B**). Cells in PDAC samples mainly consisted of CD8_C1-GNLY (Tcyt) subclusters, while CD4_C1-TCF7 (Tn) subpopulations were the predominant T cells in control samples (**Figures 3C**; **S5C**, **S6**).

Furthermore, DEG analysis of total T cells, CD4^+^ T cells or CD8^+^ T cells in PDAC versus healthy controls indicated that the level of *RACK1, ATP5F1E, FYB1, NOP53, VSIR*, and *PRF1*were increased (**Figures S7A–C**). GSEA of upregulated genes revealed that the signaling pathway, such as lymphocyte mediated immunity, leukocyte mediated cytotoxicity, immune response, and cell killing were enriched in PDAC patients (**Figures 3D**; **S7D, E**, **S8**).

To examine the differences of cytotoxic T cells between PDAC patients and healthy individuals, we reclassified T cells into two major clusters: cytotoxic cluster and non-cytotoxic cluster, according to high level of *GZMH, GZMK, PRF1, GNLY*, and *NKG7*, and low expression of *LEF1, SELL, TCF7*, and *CCR7* (**Figures 3E, F**). We found that the proportion of cytotoxic cluster in total T cells was more abundant in PDAC patients than the controls (P = 0.032, **Figures 3G, H**), with the percent of cytotoxic T cells in the control group increased from 18% to 37% of the total T cell population, whereas the median percentage of non-cytotoxic cluster in patients (50%) was less abundant than the controls (70%) (P = 0.031, **Figure 3I**). We then detected the expression of five cytotoxic genes (*NKG, PRF1, GNLY, GZMA* and *GZMB*) in each cell. As a result, most cells in non-cytotoxic cluster contained either 0 or 1 cytotoxic gene in both PDAC patients and healthy controls (**Figure 3J**, right). Cells in cytotoxic cluster contained 4 and 5 cytotoxic genes were more abundant in patients when compare with controls (**Figure 3J**, left). These findings suggested that the proportion of cytotoxic T cells and the level of cytotoxicity per cell might be more elevated in PDAC patients.

### CD8^+^ T-cell dependent unique TCR repertoire changes in pancreatic cancer

Next, we examined the TCR repertoire of the same T cells from which we extracted transcriptome data from the patients with PDAC and the healthy controls. Paired transcripts that are distally encoded but co-expressed in single cells, TCR α (TRA) and TCR β (TRB) were sequenced and filtered through a programmed filtering system (Cell ranger, 10× genomics). After barcode correction and trimming, V(D)J genes in the complementary determining region 3 (CDR3) of the TCR transcripts were annotated.

**Figure 4:**
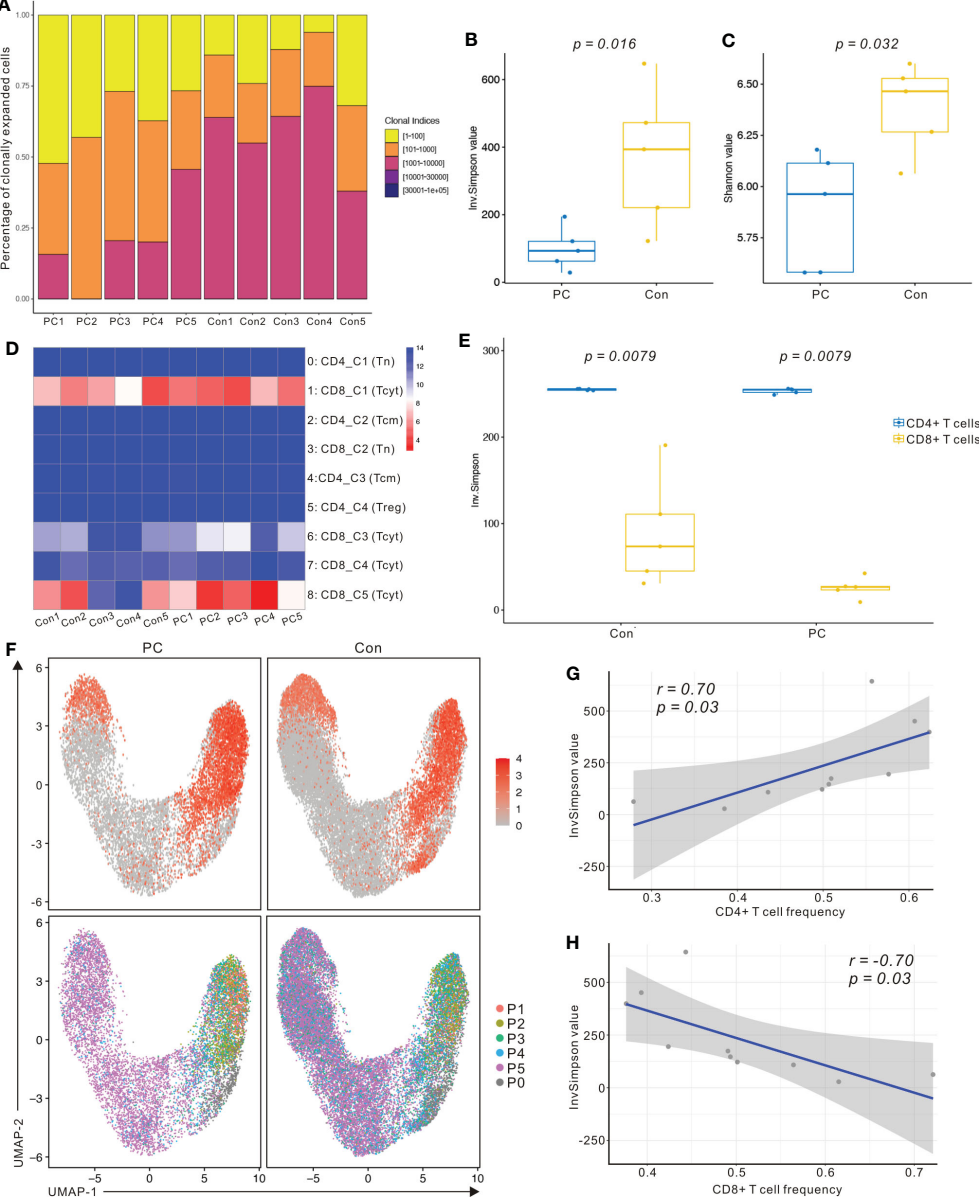
scRNA sequencing and TCR-seq reveals CD8^+^ T cell group dependent unique TCR repertoire changes in PDAC. **(A)** Percentage of clonally expanded cells in PC and control group. Yellow, cells possessing 1 to 100 expansion clonetypes; Orange, cells possessing 101 to 1000 expansion clonetypes; Fuchsia, cells possessing 1001 to 10000 expansion clonetypes; Purple, cells possessing 10001 to 30000 expansion clonetypes; Blue, cells possessing above 30001 expansion clonetypes. **(B)** InvSimpson index of PC and control group across total T cells. **(C)** Shannon–Weiner index of control and PC group across total T cells. One-way analysis of variance Tukey test was used. **(D)** Heatmap showing InvSimpson score of all T cell subclusters in each sample. **(E)** InvSimpson score of CD4^+^ T cells and CD8^+^ T cells in PC and control group. **(F)** UMAP plot of CD8A gene level in PC and control group (top). UMAP plot of TCR clonality revealing the distribution of clonally expanded cells in PC and control group (bottom). P1, cells possessing 100 to 500 expansion clones in the group; P2, cells possessing 20 to 100 expansion clones; P3, cells possessing 5 to 20 expansion clones in the group; P4, cells possessing 1 to 5 expansion clones in the group; P5, cells possessing only one expansion clone in the group; P0, unexpanded. **(G)** Correlation between InvSimpson score of T cells and CD4^+^ T-cell frequency in PC and control group. **(H)** Correlation between InvSimpson score of T cells and CD8^+^ T-cell frequency in PC and control group.

We identified a total of 27,819 unique paired αβ TCR sequences and 32484 T cells with paired TRA and TRB CDR3 sequences from the T- cell repertoire of 10 control and PDAC samples. The frequency of cells encompassing 1 to 100 expansion clonotypes was higher in the PDAC patients compared with controls (**Figure 4A**). The Inverse Simpson Diversity (InvSimpson) index was significantly lower in PDAC patients compared with the controls (**Figure 4B**) and this finding was verified by the Shannon–Weiner index (**Figure 4C**), indicating lower clonal diversity in the T cells of peripheral blood of patients with PDAC.

InvSimpson score of the distinct T- cell populations revealed CD8^+^ T cell skewed clonal diversity in the peripheral blood of patients with PDAC and healthy controls (**Figure 4D**). When we divided T cells into CD8^+^ T cells and CD4^+^ T cells, the InvSimpson score of CD8^+^ T cells was significantly lower than that of CD4^+^ T cells (**Figure 4E**). This result was further confirmed by comparing UMAPs of CD8A gene level and clonally expanding the cell populations (**Figure 4F**). Finally, we observed that InvSimpson of subjects in all samples had a negative correlation with CD8^+^ T- cell frequency, while had a positive correlation with CD4^+^ T- cell frequency (**Figures 4G, H**). This result suggests that expansion of CD8+ T cells contributes to the lower InvSimpson index and clonal diversity in patients with PDAC compared to the healthy controls, consistent with our scRNA-seq data.

**Figure 5:**
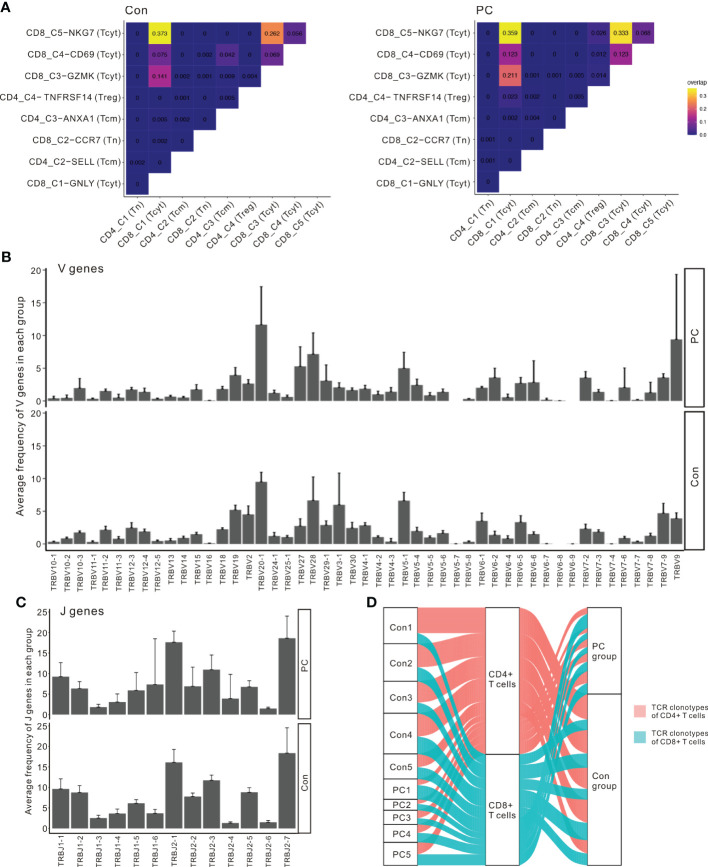
Interactions between T cell clusters and shared TCR clonotypes in PDAC. **(A)** Clonotype overlap coefficients between subclusters in PC and control group. **(B)** The frequency histogram of the 49 V genes in PC and control group. **(C)** The frequency histogram of the 13 J genes in PC and control group. **(D)** Alluvial plots tracking the frequencies of TCR clonotypes from each sample.

To investigate the interactions among the T cell clusters, we matched single-cell TCR-αβ profiling and tested the unique clonotype overlaps among subclusters from all samples (**Figure 5A**). The greatest clonotype overlaps were discovered in CD8_C5−NKG7 (Tcyt), CD8_C4-CD69, and CD8_C3−GZMK (Tcyt), especially in the PDAC samples (**Figure 5A**). This result suggests unique interactions between T cell clusters and shared TCR clonotypes in patients with PDAC. To compare the frequency of the V and J gene of the TCR, a usage frequency histogram was generated according to common usage frequency of the V and J gene (**Figures 5B, C**). V genes, including TRBV20-1 and TRBV9, and J genes, including TRBJ1-6, showed a higher frequency in patients with PDAC compared to the healthy controls. Finally, we tracked the TCR clonotypes based on the scTCRseq data and observed that the percentages of clonal TCRs were decreased in PDAC patients. (**Figure 5D**). These scTCR-seq data unveiled a visible T cell immune response in patients with PDAC which is consistent with our scRNA-seq results.

### Expansion of S100A9^+^ monocytes in patients with pancreatic ductal adenocarcinoma

**Figure 6:**
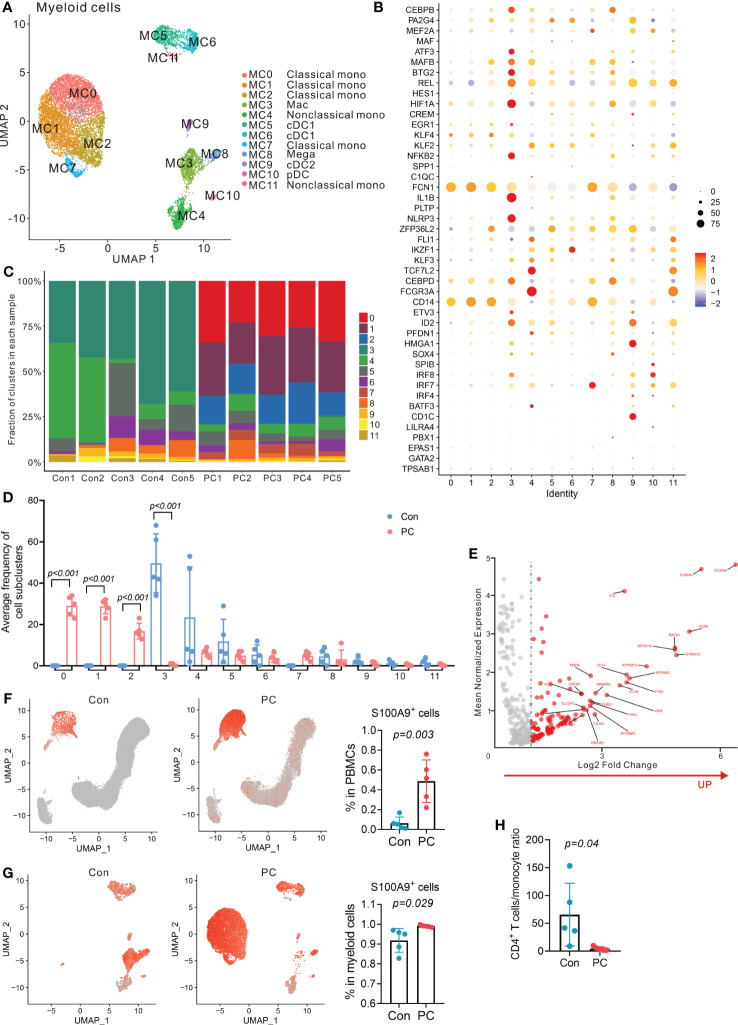
Distinct myeloid cells transcriptional signatures in PDAC and control group. **(A)** UMAP plot of myeloid cells presenting 12 clusters (mono, monocytes; Mac, macrophages; cDC, conventional dendritic cells; pDC, plasmacytoid dendritic cells; Mega, megakaryocytes). **(B)** Dot plot depicting percent expression and average expression of canonical marker genes in all myeloid cell subclusters. **(C)** Fraction of 12 myeloid cell subclusters in each sample from PC patient and control. **(D)** Average proportion of myeloid cell subclusters in PC and control group. One-way analysis of variance Wilcoxon rank sum test was used. **(E)** Analysis of differentially expressed genes in monocytes from PC versus control group. Red dots represent the significantly upregulated top 25 genes in PC versus control group. **(F)** UMAP plot of S100A9 gene level in total PBMCs from control and PC group (left and middle). Proportion of S100A9+ cells in total PBMCs from PC and control group (right). One-way analysis of variance Wilcoxon rank sum test was used. **(G)** UMAP plot of S100A9 gene level in myeloid cells from control and PC group (left and middle). Proportion of S100A9+ cells in myeloid cells from PC and control group (right). One-way analysis of variance Wilcoxon rank sum test was used. **(H)** CD4^+^ T cells/monocyte ratio in PC and control group.

Myeloid cells from peripheral blood or tumor microenvironment play key roles in many types of cancer (24), including pancreatic cancer (25). Previous result showed that myeloid cell numbers were significantly increased in patients with PDAC compared with the controls (P = 0.016, **Figure 1G**). We further clustered the myeloid cells into 12 distinct subtypes (**Figure 6A**). Myeloid cell clusters were manually annotated by the typical cell marker gene expression (26, 27) (**Figures 6B**; **S9A, B**). MC0, MC1, MC2, and MC7 correspond to classical monocytes due to the presence of *CD14, FCN1, SELL, CD36, S100A8, S100A12*, and *MS4A6A*. MC4 and MC11 correspond to nonclassical monocytes according to the expression of *FCGR3A, FCGR3B, FCN1, TCF7L2, CDKN1C*, and *CDH23*. MC3 corresponds to macrophages (Mac), characterized by expression of *NLRP3, IL1B, CD83, CCL3L3, THBS1*, and *PLAUR*. MC5 and MC6 belong to type 1 conventional dendritic cells (cDC1), identified by the presence of *CST7, IL6ST, IFITM1*, and *ANXA6*. MC9 belongs to type 2 cDCs (cDC2) due to the expression of *CD1C, CLEC10A, FCGR2B, ADAM8, FCER1A*, and *ADAM28*. MC10 corresponds to plasmacytoid DCs (pDC) due to the presence of *IRF7, IRF8*, and *SPIB*. MC8 corresponds to megakaryocytes (Mega), characterized by presence of *PPBP* and *PF4*.

Importantly, we found that distinct subclusters contributed to the myeloid compartment of peripheral blood immune cells (**Figure 6C**). The frequency of classical monocytes (MC0, MC1, MC2 and MC7) was significantly higher in patients with PDAC than the controls, while macrophages (MC3) comprised a higher proportion of myeloid cells in the peripheral blood of healthy controls than the PDAC patients (**Figure 6D**).

Furthermore, DEG analysis of total myeloid cells or monocyte subpopulations in PDAC patients versus healthy controls revealed that the expression of *S100A9, S100A8, S100A12, RACK1, LYZ*, and *VCAN* was upregulated (**Figures 6E**; **S10**). These data indicated that PDAC patients had a higher expression of S100A family members, which was found to be associated with tumor aggressive and metastasis previously (28). Up-regulation of S100A9 expression inhibited the differentiation of DCs and macrophages and induced accumulation of MDSCs (29). We found that the proportion of S100A9^+^ monocytes was significantly up-regulated in PDAC patients compared to the healthy controls (**Figures 6F, G**). We also found that PDAC patients had a lower CD4^+^ T cells/monocyte ratio than that of healthy controls (**Figure 6H**). High S100A9 levels in peripheral blood monocytes and a lower ratio of CD4^+^ T cells/monocyte was previously found to correlate positively with poor response to anti-PD-1 immunotherapy but inversely with overall survival in melanoma patients (30). Overall, single-cell analysis of PBMCs revealed a significantly enriched S100A9^+^ monocyte population in PDAC patients and the potential role of S100A9^+^ monocytes in immunotherapy resistance.

### The expression of *HAVCR2* (TIM-3) in PDAC

**Figure 7:**
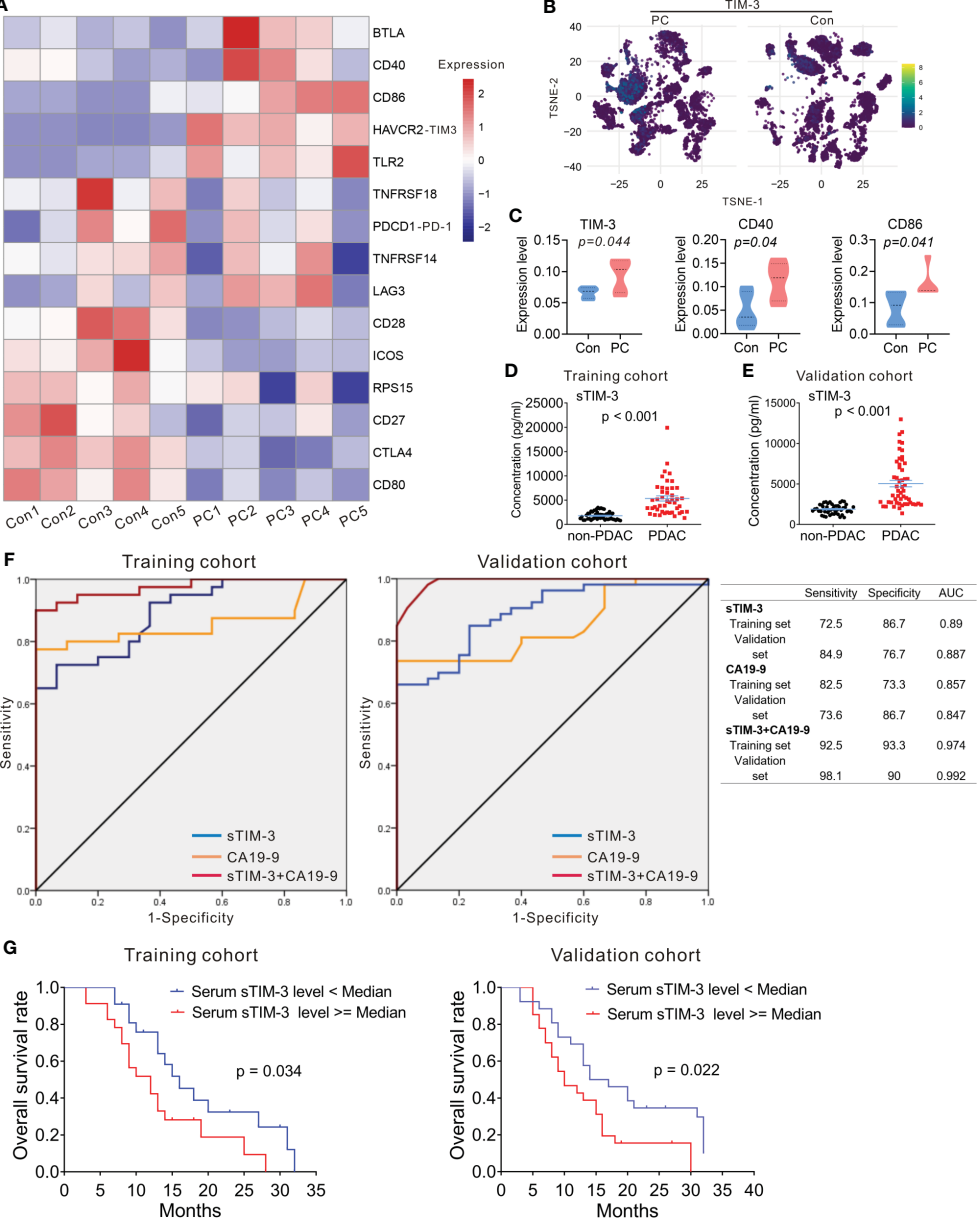
Expression of *HAVCR2* (TIM-3) in PDAC. **(A)** Heatmap displaying expression of select genes in each sample from PC and control group. **(B)** T-SNE plot showing the selected marker TIM-3 in PC and control group. **(C)** Protein expression level of TIM-3 (left), CD40 (middle), and CD86 (right) in PC and control group. One-way analysis of variance Wilcoxon rank sum test was used. **(D, E)** Serum levels of sTIM-3 in PDAC patients and non-PDAC participants. The p-values were calculated based on a Student’s t-test. **(F)** The ROC curves of sTIM-3, CA19-9, and sTIM-3+CA19-9 for PDAC detection in the training cohort and validation cohort. Table showing the sensitivity, specificity and AUC value of the three models in these two cohorts. **(G)** Kaplan–Meyer plot of OS in PDAC patients with high or low serum sTIM-3 expression in different cohorts. AUC, area under the curve; CA19-9, carcinoma antigen 19-9; ROC, receiver operating characteristic.

Immune checkpoint proteins have been associated with overall survival (OS) and progression in cancer patients as previously reported (31). We then compared mRNA expression of immune checkpoint proteins between PDAC patients and controls using scRNA-seq. We found that RNA expression level of *BTLA, CD40, CD86, HAVCR2* (TIM-3), and *TLR2* were increased in PDAC compared to controls, while the level of *CD28, ICOS, CTLA4*, and *CD80* were decreased (**Figure 7A**). Moreover, we examined protein level of TIM-3, LAG3, PD-1, GITR, TIGIT, CD274 (PD-L1), CD40, CD86, CD27, CD28, ICOS, CTLA4, and CD80 in both PDAC and controls by mass cytometry (**Figures 7B, C**; **S11**, **S12**), and found that protein level of TIM-3, CD40, and CD86 in immune cells was higher in PDAC patients than that of the controls (**Figure 7C**), consistent with our scRNA-seq results.

Previous studies had revealed that serum levels of soluble protein detected in the serum of PDAC patients had prognostic value (32). We next measured the serum levels of immune checkpoint proteins of the peripheral blood in 45 PDAC patients and 50 non-PDAC participants (including 20 participants with benign pancreatic diseases and 30 healthy controls). The clinicopathological characteristics of 45 patients with PDAC and 50 non-PDAC participants are presented in **Table S6**. Sixteen soluble immunologic protein (including sBTLA, sCD27, sCD28, sTIM-3, sHVEM, sCD40, sGITR, sLAG-3, sTLR-2, sGITRL, sPD-1, sCTLA-4, sCD80/B7-1, sCD86/B7-2, sPD-L1, and sICOS) levels were detectable in all serum samples. Median values are presented in **Table S7**. There was no significant difference in sex and age distribution between the PDAC patients and the non-PDAC participants (**Table S6**). The level of serum sTIM-3 was significantly higher in PDAC patients, compared to the non-PDAC participants (**Figure 7D**; **Table S7**). The levels of other soluble proteins were no difference between PDAC cohort and non-PDAC participants (**Table S7**).

To confirm whether sTIM-3 can be a potentially valuable diagnostic marker, the cohort contained 45 PDAC patients and 50 non-PDAC participants were defined as the training cohort, and another 53 PDAC patients, 22 participants with benign pancreatic diseases and 25 healthy volunteers from our center were defined as the validation cohort. The clinicopathological characteristics of all participants in validation cohort are presented in **Table S8**. In the validation cohort, the serum sTIM-3 level of the patients with PDAC was significantly higher than that of the non-PDAC subjects (**Figure 7E**), consistent with the results observed in the training cohort. sTIM-3 alone achieved a sensitivity of 72.5% and a specificity of 86.7%, with an AUC of 0.89 in the training cohort (**Figure 7F**). In the validation cohort, the sensitivity was 84.9% and the specificity was 76.7%, with an AUC of 0.887 (**Figure 7F**). We then combined the CA19-9 level and sTIM-3 together to generate a new diagnostic model (named as sTIM-3+CA19-9), which had a better sensitivity of 92.5% and specificity of 93.3% in the training cohort, with an AUC of 0.974, which was greater than CA19-9 alone (AUC=0.857). Similarly, the sensitivity (98.1%) and specificity (90%) in the validation cohort were also enhanced compared with the sTIM-3 alone (**Figure 7F**). The AUC value of sTIM-3+CA19-9 in the validation cohort was 0.992, which was better than that of CA19-9 (AUC=0.847) or sTIM-3 alone (AUC=0.887). These results indicated that sTIM-3 combined the CA19-9 may be of value in the diagnosis and follow-up for patients with PDAC.

**Figure 8:**
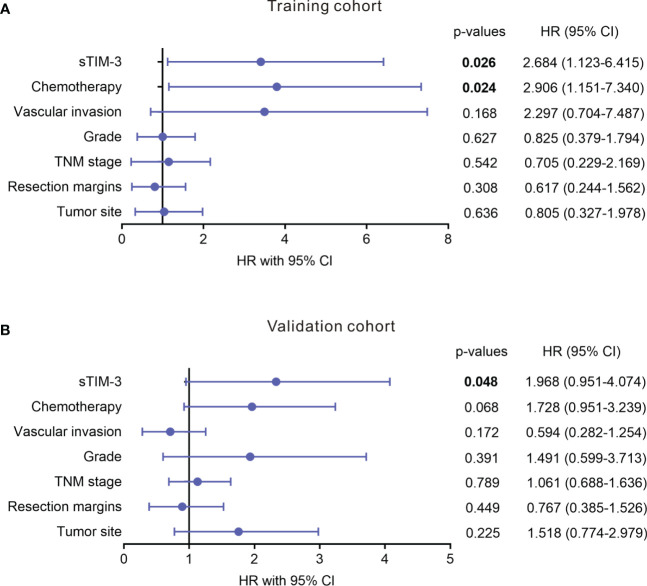
Multivariate analysis of level of serum soluble TIM-3 for association with OS in patients with PDAC from different cohorts. **(A)** Training cohort. **(B)** Validation cohort. Analysis was adjusted for tumor site, resection margins, TNM stage, grade, vascular invasion, and chemotherapy.

To determine the prognostic values of sTIM-3, we performed Kaplan-Meier analysis and found that PDAC patients with high serum sTIM-3 level had worse OS in the training cohort (Cutoff value = median, p = 0.034, **Figure 7G**) or in the validation cohort (Cutoff value = median, p = 0.022, **Figure 7G**). We then performed multivariate Cox regression analysis to determine if sTIM-3 remains an independent predictor of OS. As shown in **Figures 8A, B**, either in the training cohort or in the validation cohort, the correlation with OS remains statistically significant for sTIM-3 after adjusting for tumor site, resection margins, TNM stage, grade, vascular invasion, and postoperative chemotherapy.

## Discussion

We have shown that there were increased proportion of myeloid cells as well as increased proportion of cytotoxic T cells in the peripheral blood of patients with PDAC, suggesting the presence of a certain degree of systemic inflammatory and immune response in patients with PDAC, rather than a state of complete immunologically “cold” tumor. We also found increased S100A expression on the monocytes of patients with PDAC. S100A proteins have been found to be associated with cancer progression. In addition, we found increased level of sTIM-3 in the peripheral blood of patients with PDAC and that high sTIM-3 serum level correlated with worse OS, suggesting that serum level of immune checkpoint proteins may be critical for mounting appropriate immune response for patients with PDAC.

The mechanism of treatment resistance of cancer is determined by several factors including intrinsic characteristics of tumor cells, systemic immune state of a host and tumor microenvironment (33) (34). Studies have shown that higher TCR clonal diversity of PD-1^+^ CD8^+^ T cells provides enhanced opportunities for tumor neoantigen recognition (35). In patients with non-small cell lung cancer the clonal diversity of PD-1^+^ CD8^+^ TCR in peripheral blood correlated with better survival (35, 36). It is not clear if such a T cell clonal diversity is associated with the survival of PDAC. Our high-dimensional sequencing to simultaneously assess TCR repertoire and RNA/protein expression at the single-cell level provide a more comprehensive picture for understanding the immune state of patients with PDAC.

TCR diversity plays an important role in maintaining immune system homeostasis, and the loss of TCR clonal diversity may lead to diseases (37). In the TCR repertoire analysis, patients with PDAC had significantly different T cell characteristics compared with the healthy control. TCR diversity in PDAC was significantly reduced. Peripheral blood T cells from PDAC patients show increased clonal expansion in CD8^+^ T cell subsets, and higher expansion of CD8^+^ T cells contributes to the lower clonal diversity in PDAC. This may be due to the specific immune response of peripheral blood T cells to some tumor-specific antigens and the antigen-dependent selective rearrangement of CD8^+^ TCR. Our data suggest that the different TCR diversity found in control versus PDAC are highly correlated with the distribution of T-cell subsets. Although monoclonal expansion of peripheral blood toxic CD8^+^ TCR in PDAC patients has been identified, the specific antigens responsible for clonal changes in T cells require further perform epitopes matching analysis and validation study.

Myeloid cells from the peripheral blood or tumor microenvironment play a key role in malignancies including immune evasion. Previous study found increased number of CD14^+^ S100A9^high^ myeloid cells in the peripheral blood of colon cancer patients compared to healthy individuals (38). Also, it has been shown that high numbers of S100A9^+^ inflammatory cells in tumor stroma correlated with shorter survival in patients with prostate cancer (39). Moreover, in patients with gastric cancer high plasma levels of S100A8/A9 correlated with the increased population of myeloid cells (40). Our results revealed that proportions of the myeloid cells from the peripheral blood were significantly increased in patients with PDAC and that increased S100A9^+^ monocytes and lower ratio of CD4^+^ T cells/monocytes may be partly responsible for poor response to anti-PD-1/PD-L1 therapy.

Recent studies showed that TIM-3 and galectin-9 interaction was involved in the immune escape of several malignancies such as acute myeloid leukemia (AML) and non-small cell lung cancer (41, 42). Soluble TIM-3 level was highly increased in the blood plasma of AML patients and was shown to prevent secretion of interleukin-2 (IL-2) required for the activation of NK cells and cytotoxic lymphoid cells (41). The plasma level of TIM-3 reportedly could be used to predict failure of graft-versus-host-disease (GVHD) treatment and mortality (43). However, serum soluble TIM-3 has not yet been investigated in PDAC. Our results suggest that serum sTIM-3 could be an additional valuable biomarker for the diagnosis and prognosis of PDAC. Although the trade-off between sensitivity and specificity depends on the clinical setting, it is worth noting that the combination of sTIM-3 and CA19-9 further improved the AUC value, suggesting that sTIM-3 is complementary to this traditional tumor marker, resulting in excellent performance. And that high serum sTIM-3 level correlated with poor outcomes in patients with PDAC by univariate and multivariate analysis.

In conclusion, our comprehensive single-cell RNA-seq data provides an in-depth understanding of the immune signatures of the peripheral blood of patients with PDAC. The expansion of peripheral blood cytotoxic CD8^+^ T cells in PDAC patients resulted in a decrease of TCR clonal diversity, suggesting that PDAC patients may retain a specific immune response to potential novel tumor antigens in PDAC that could be valuable for therapeutic targeting.

## Data availability statement

The data presented in the study are deposited in the GEO repository, accession number GSE87916, and in the BioProject repository, accession number PRJNA348194.

## Ethics statement

The studies involving human participants were reviewed and approved by the Committee for the Ethical Review of Research, Fujian Medical University Union Hospital (2020WSJK055). The patients/participants provided their written informed consent to participate in this study.

## Author contributions

HH, FL, and YP conceived the research. YP, JG and JL designed the methodology. YP performed the experiments. JG and JL performed the data analysis. YM, ZH, YL, and SW assisted with experiments. YP wrote the original draft of the manuscript. MP, FL and HH reviewed and edited the manuscript. HH and FL supervised the study. All authors contributed to the article and approved the submitted version.
