# Supplementary material for: High-dimensional single-cell analysis unveils distinct immune signatures of peripheral blood in patients with pancreatic ductal adenocarcinoma
Source: Front Endocrinol (Lausanne). 2023 Jun 6;14:1181538. doi: 10.3389/fendo.2023.1181538 (PMC10281055; doi:10.3389/fendo.2023.1181538)
Supplement: Supplementary file 1 [file Image_1.pdf]

High-dimensional Single-cell analysis Unveils Distinct Immune Signatures of Peripheral Blood in Patients with Pancreatic Ductal Adenocarcinoma

Yu Pan[1#], Jianfeng Gao[1#], Jiajing Lin[1#], Yuan Ma[1], Zelin Hou[1], Yali Lin[1], Shi Wen[1], Minggui Pan[2], Fengchun Lu[1*], Heguang Huang[1*]

Supplementary Materials
(Figure S1 – S12)

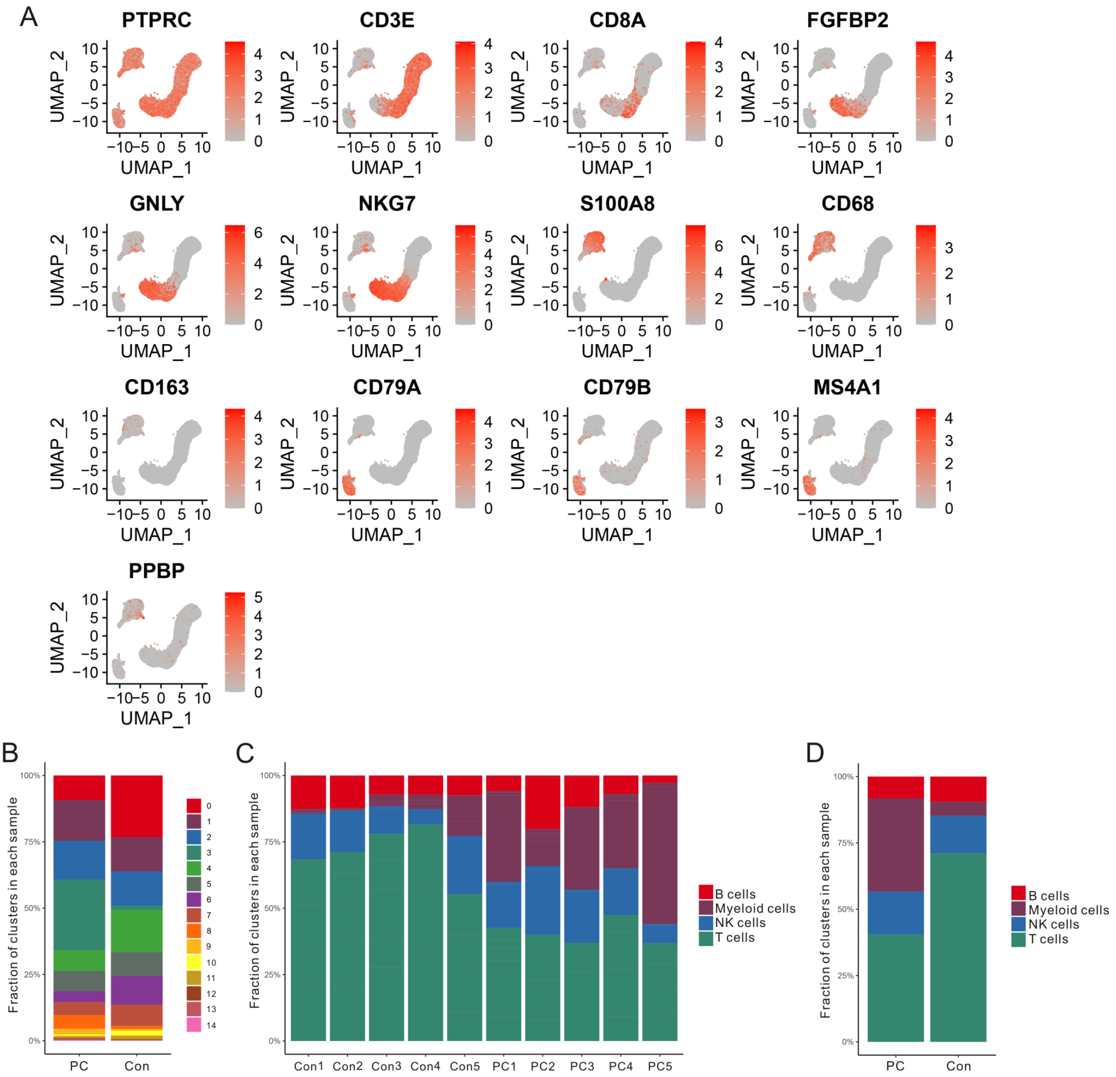

Figure S1 scRNA-seq of PBMCs in PC and control group. (A) UMAP plot of immune cells displaying marker gene expression. (B) Fraction of 15 immune clusters in PC and control group. (C-D) Proportion of immune subclusters in PC and control group.

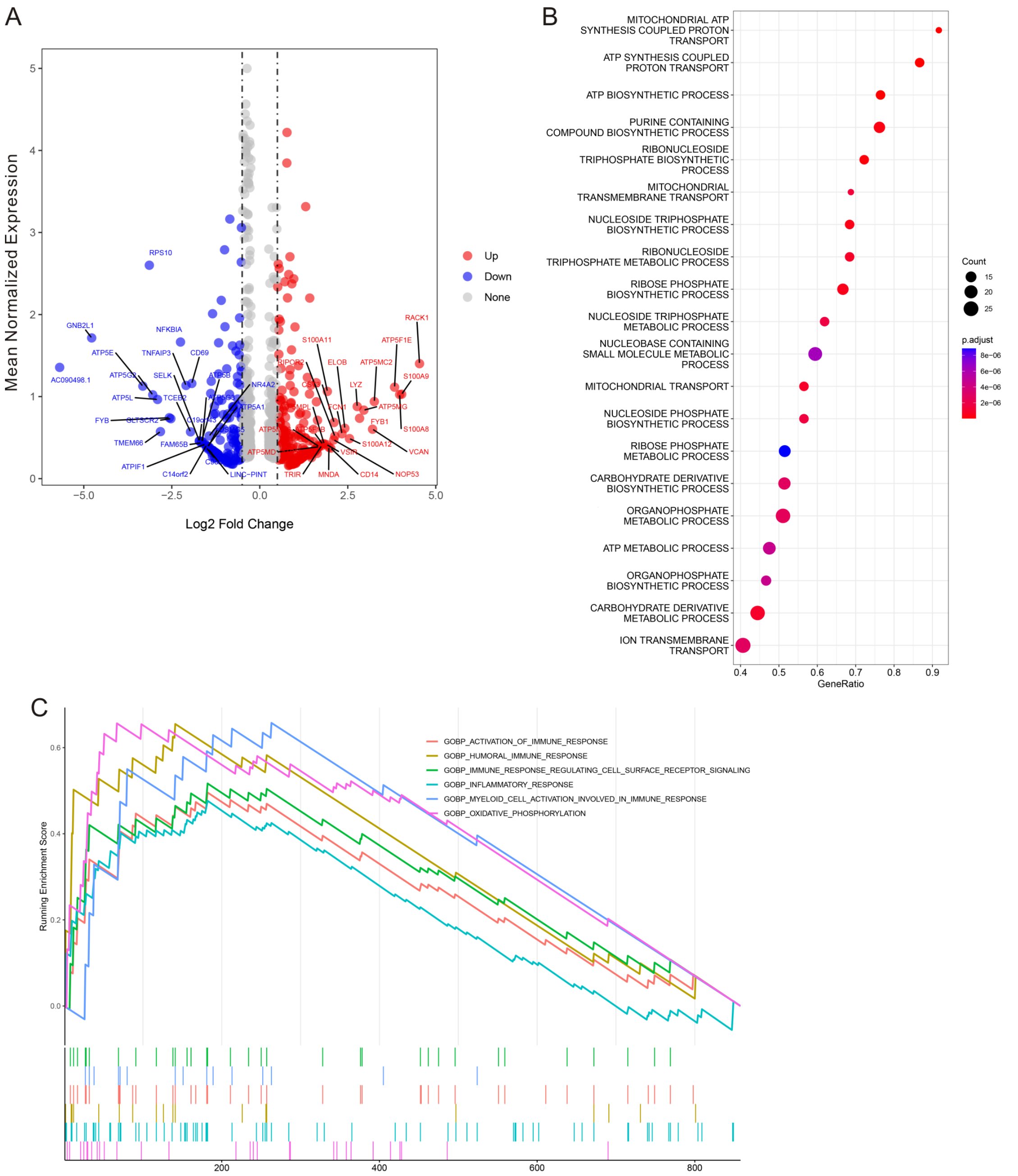

Figure S2 DEGs and functional enrichment analysis of PBMCs from PC and control. (A) Analysis of differentially expressed genes in PBMCs from PC versus control group. Red dots represent the significantly upregulated top 25 genes in PC versus control group. Blue dots represent the significantly downregulated top 25 genes in PC versus control group. (B) Functional enrichment analysis displaying biological processes enriched in PC versus control. (C) Gene set enrichment analysis of differentially expressed genes in total PBMCs from PC versus control group.

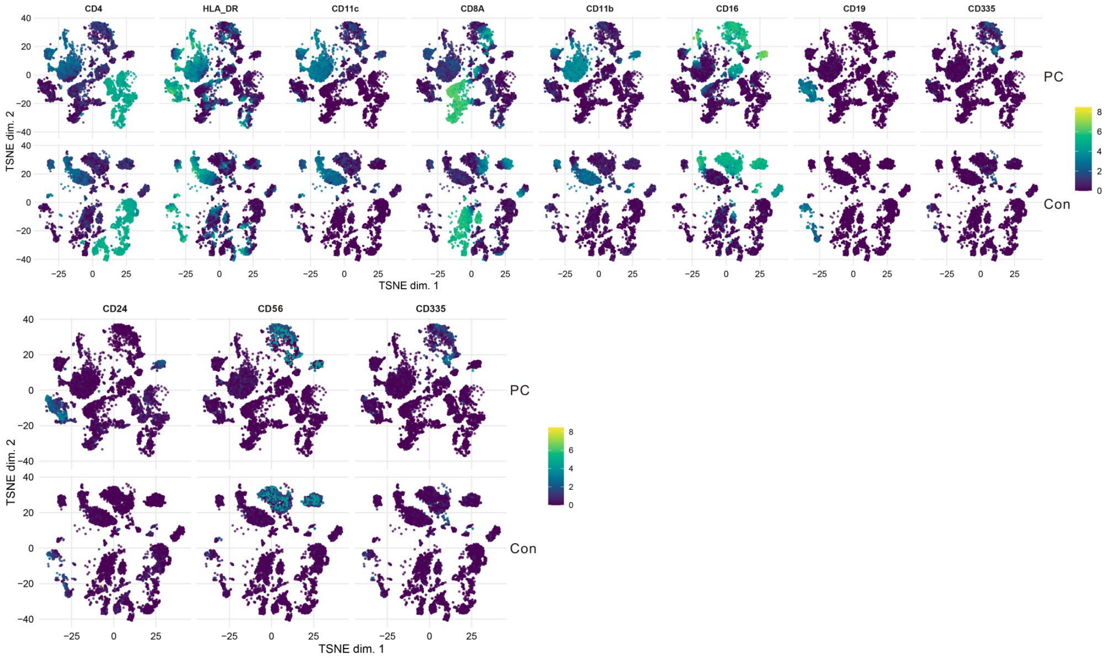

Figure S3 T-SNE plot of mass cytometry showing the selected markers in PC and control group.

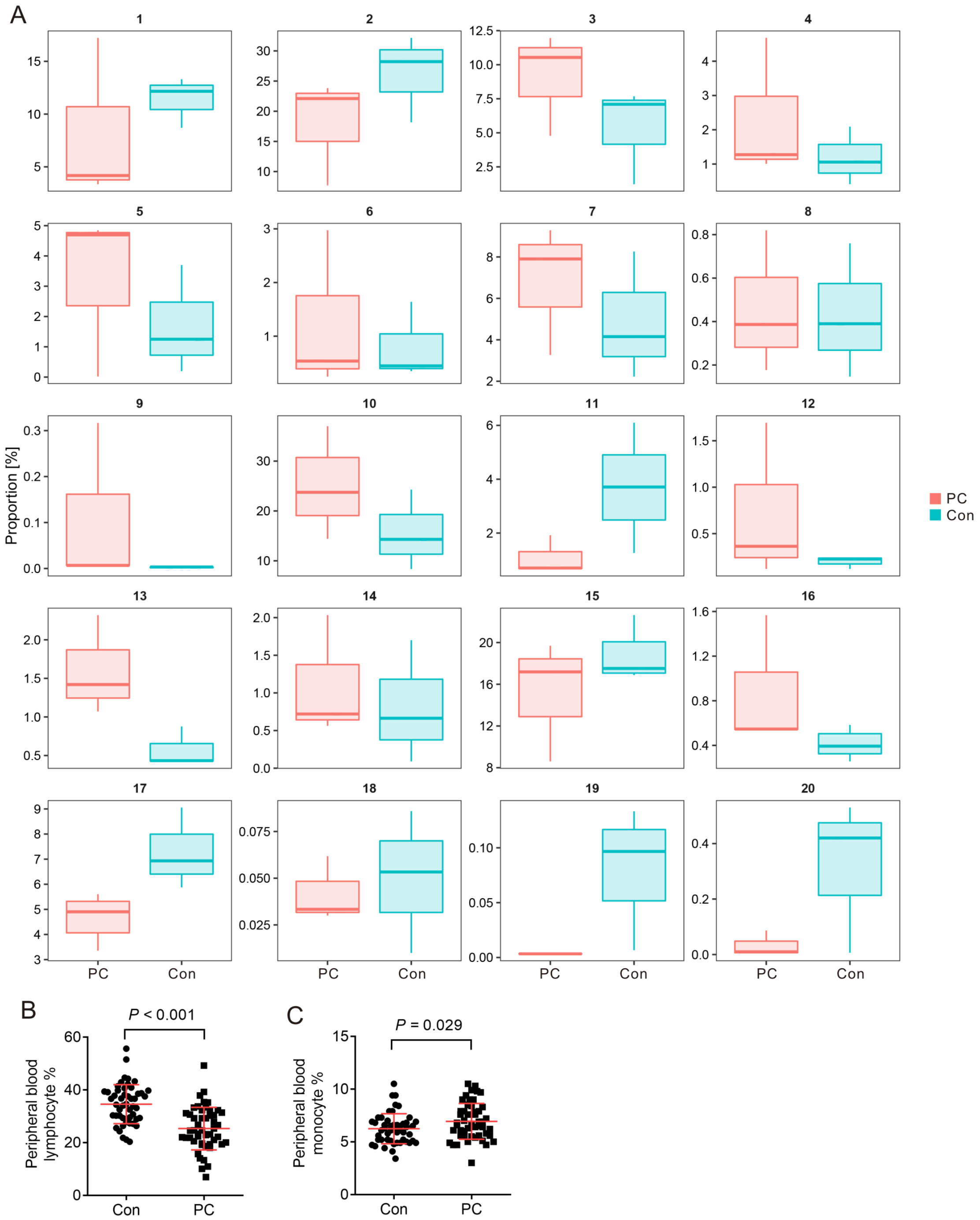

Figure S4 Mass cytometry showed the subclusters of PBMCs in PC and control group. (A) The cell frequency of each cluster revealed by mass cytometry. (B) Proportion of peripheral blood lymphocytes in PC and Con group. (C) Proportion of peripheral blood monocytes in PC and Con group.

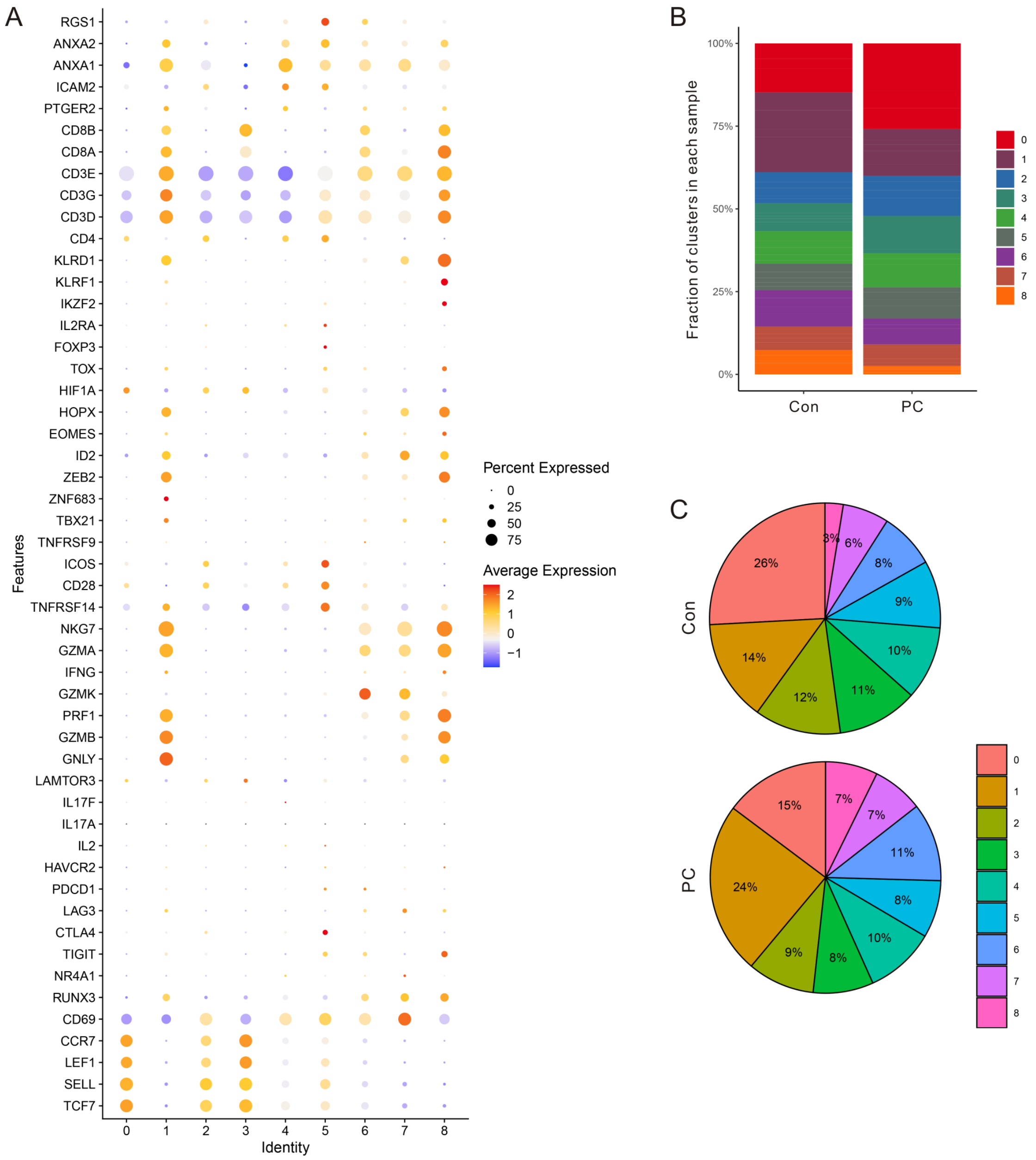

Figure S5 T-cell transcriptional signatures in PC and control. (A) Dot plot depicting percent expression and average expression of canonical marker genes in T cell subclusters. (B) Fraction of 9 T cells subclusters in PC and control group. (C) Average proportion of T cells subclusters in PC and control group.

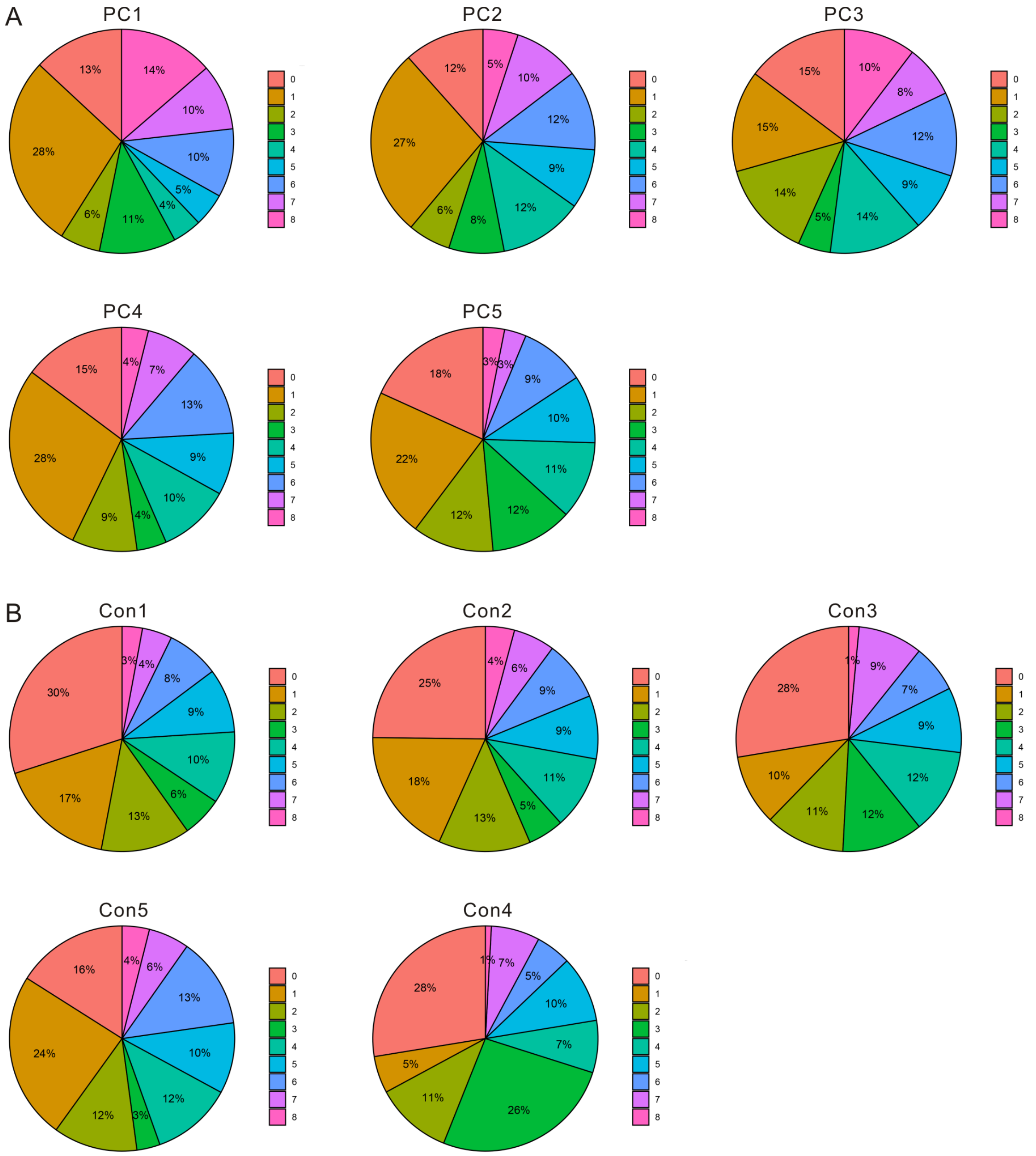

Figure S6 Proportion of T cells subclusters in each sample from PC and control group. (A) Samples from PC group. (B) Sampales from control group.

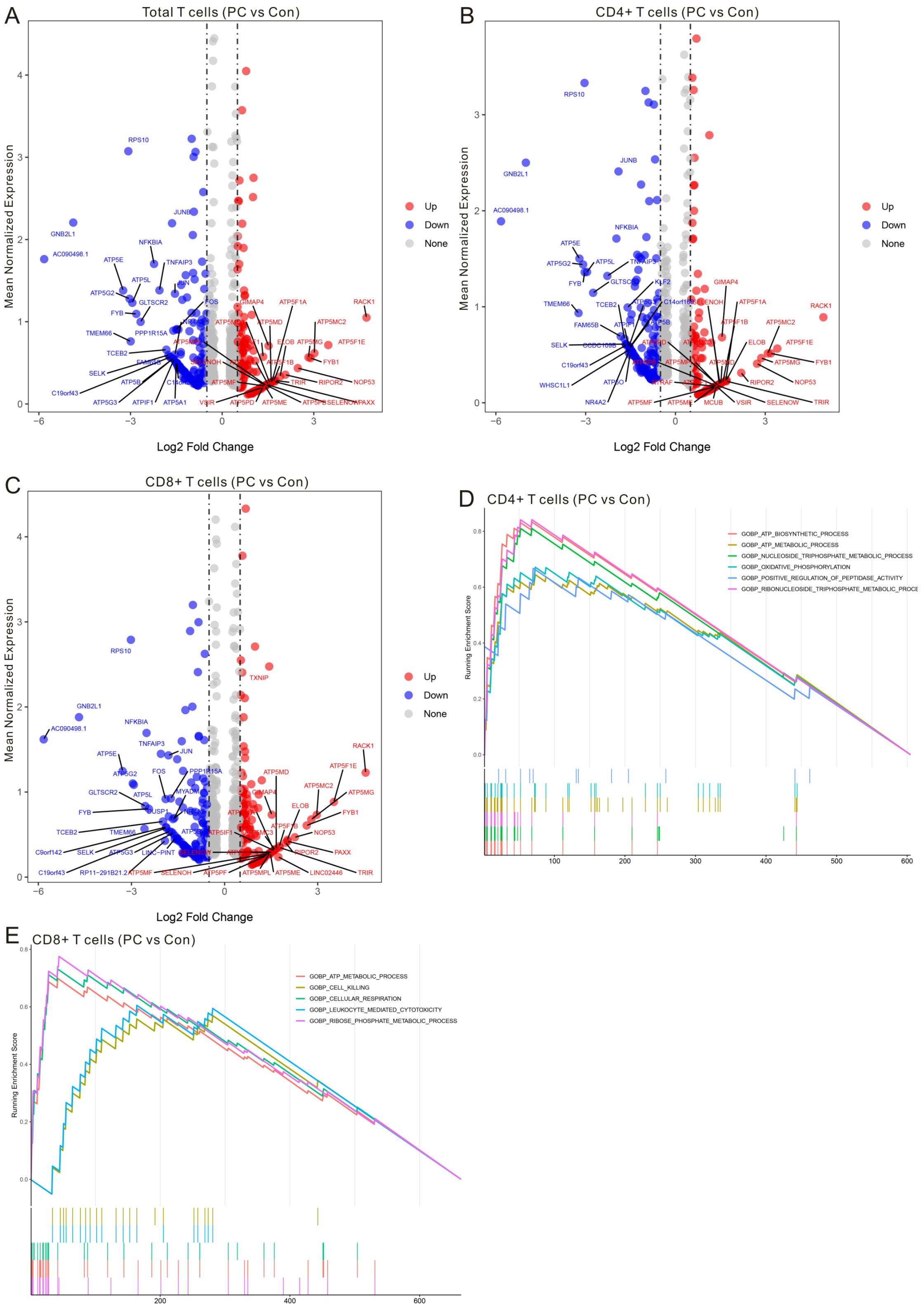

Figure S7 DEGs and functional enrichment analysis of T cells from PC and control. (A) Analysis of differentially expressed genes in total T cells from PC versus control group. (B) Analysis of differentially expressed genes in CD4^{+} T cells from PC versus control group. (C) Analysis of differentially expressed genes in CD8^{+} T cells from PC versus control group. Red dots represent the significantly upregulated top 25 genes in PC versus control group. Blue dots represent the significantly downregulated top 25

genes in PC versus control group. (D) Gene set enrichment analysis of differentially expressed genes in $CD4^+$ T cells from PC versus control group. (E) Gene set enrichment analysis of differentially expressed genes in $CD8^+$ T cells from PC versus control group.

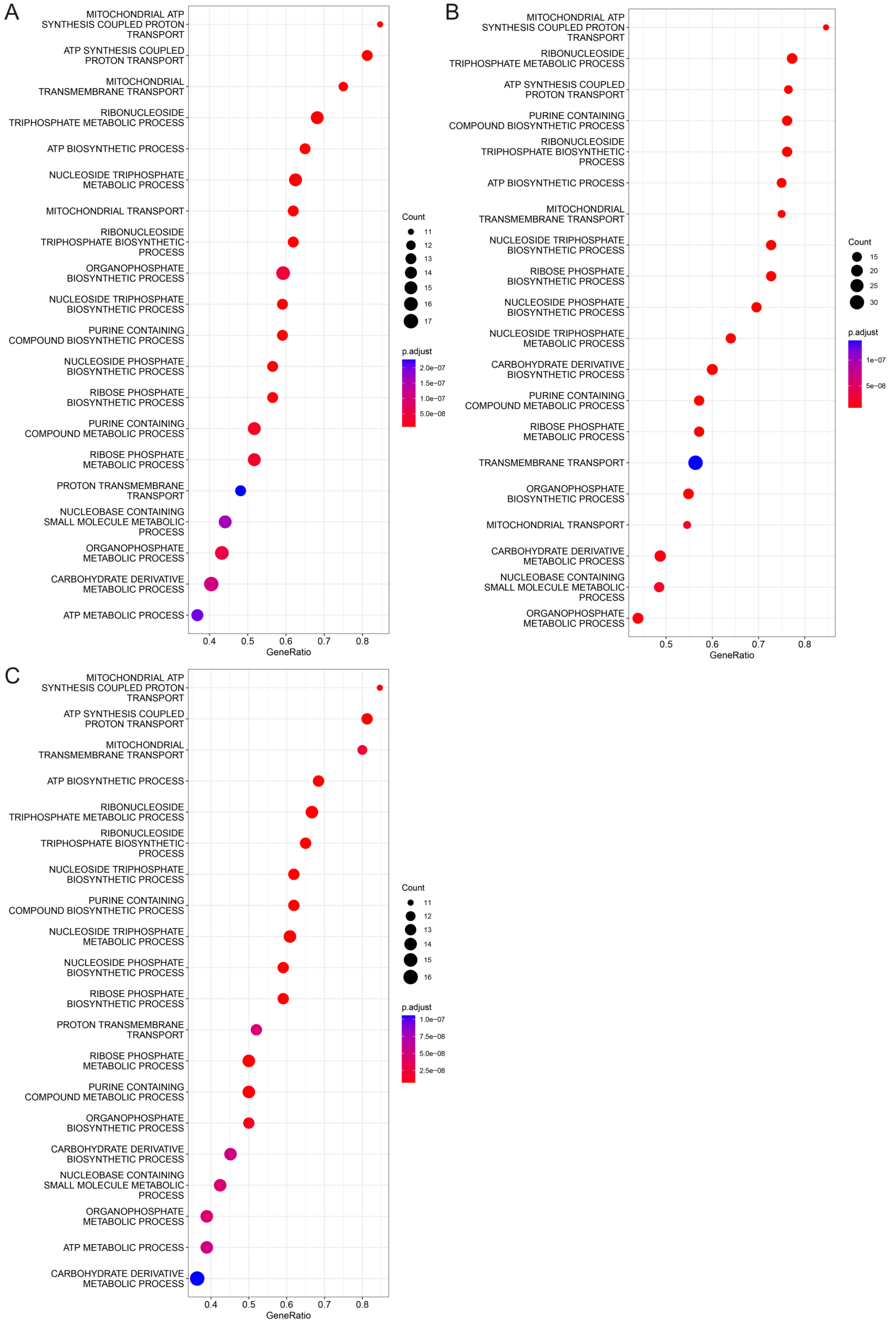

Figure S8 Functional enrichment analysis of T cells from PC and control. (A) Functional enrichment analysis of total T cells displaying biological processes enriched in PC versus control. (B) Functional enrichment analysis of $CD4^+$ T cells displaying biological processes enriched in PC versus control. (C) Functional

enrichment analysis of CD8^{+} T cells displaying biological processes enriched in PC versus control.

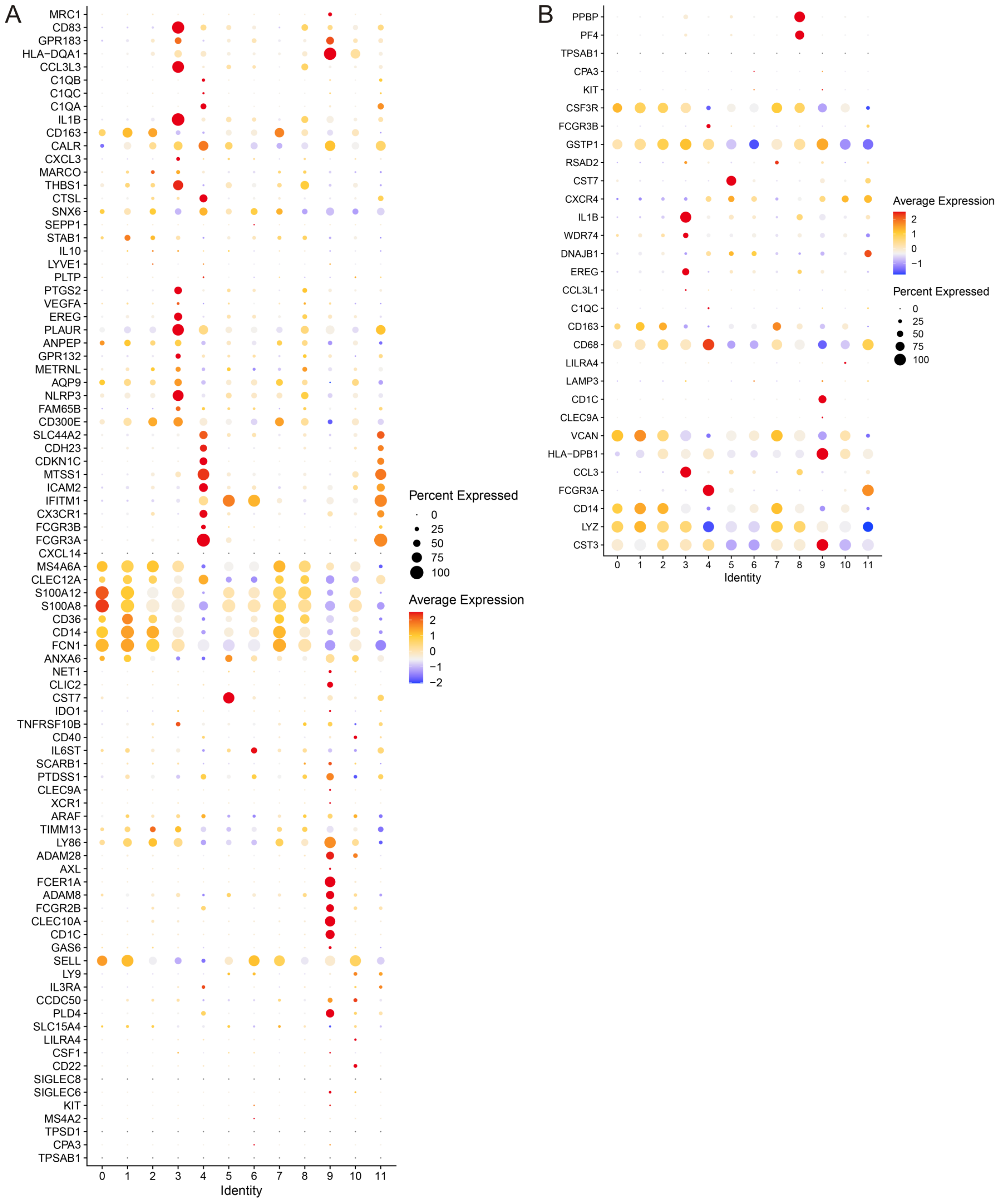

Figure S9 Myeloid-cell transcriptional signatures in PC and control. (A-B) Dot plot depicting percent expression and average expression of canonical marker genes in Myeloid cell subclusters.

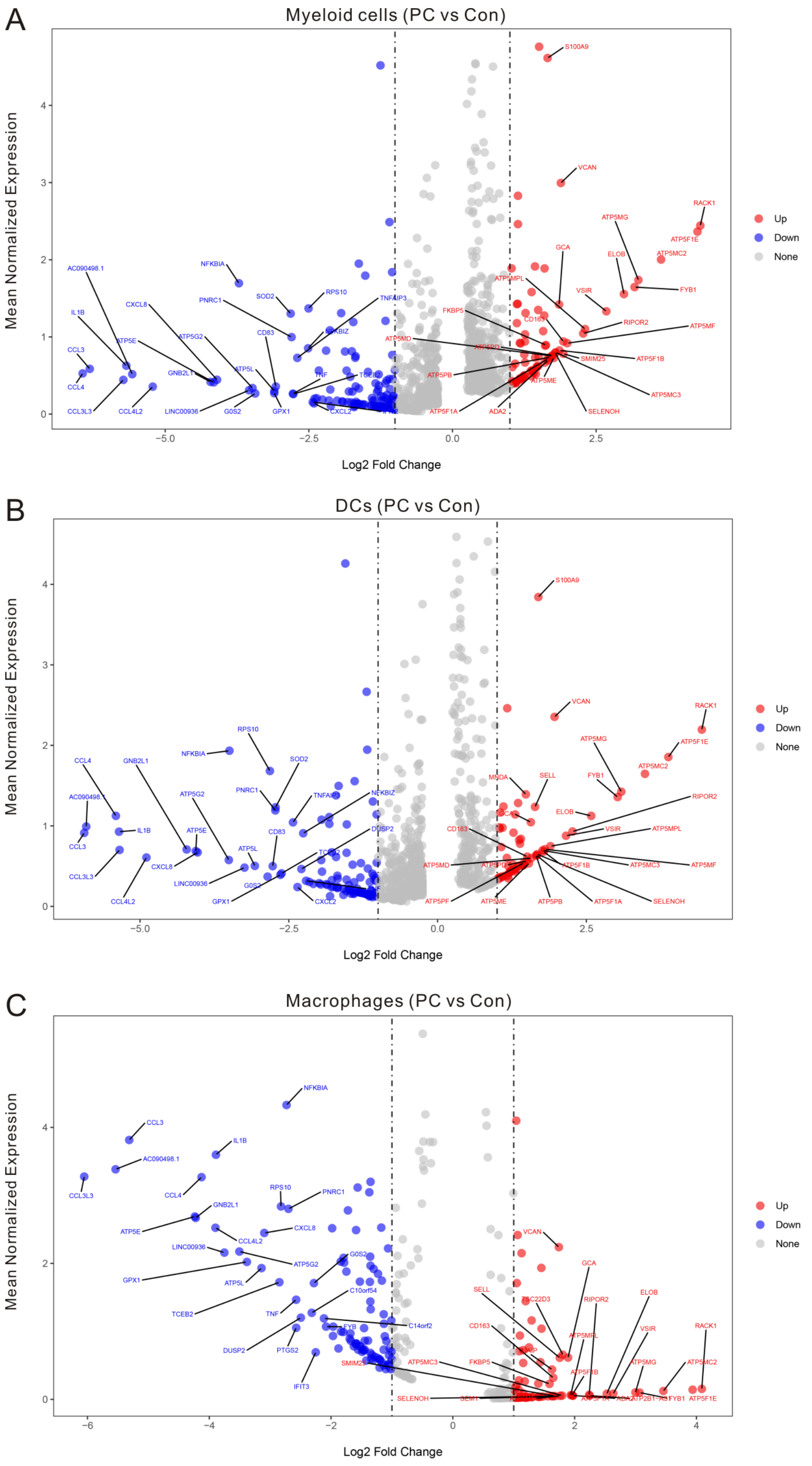

Figure S10 DEGs analysis of myeloid cells from PC and control. (A) Analysis of DEGs in total myeloid cells from PC versus control group. (B) Analysis of DEGs in DCs from PC versus control group. (C) Analysis of DEGs in macrophages from PC versus control group. Red dots represent the significantly upregulated top 25 genes in PC versus control group. Blue dots represent the significantly downregulated top 25 genes in PC versus control group.

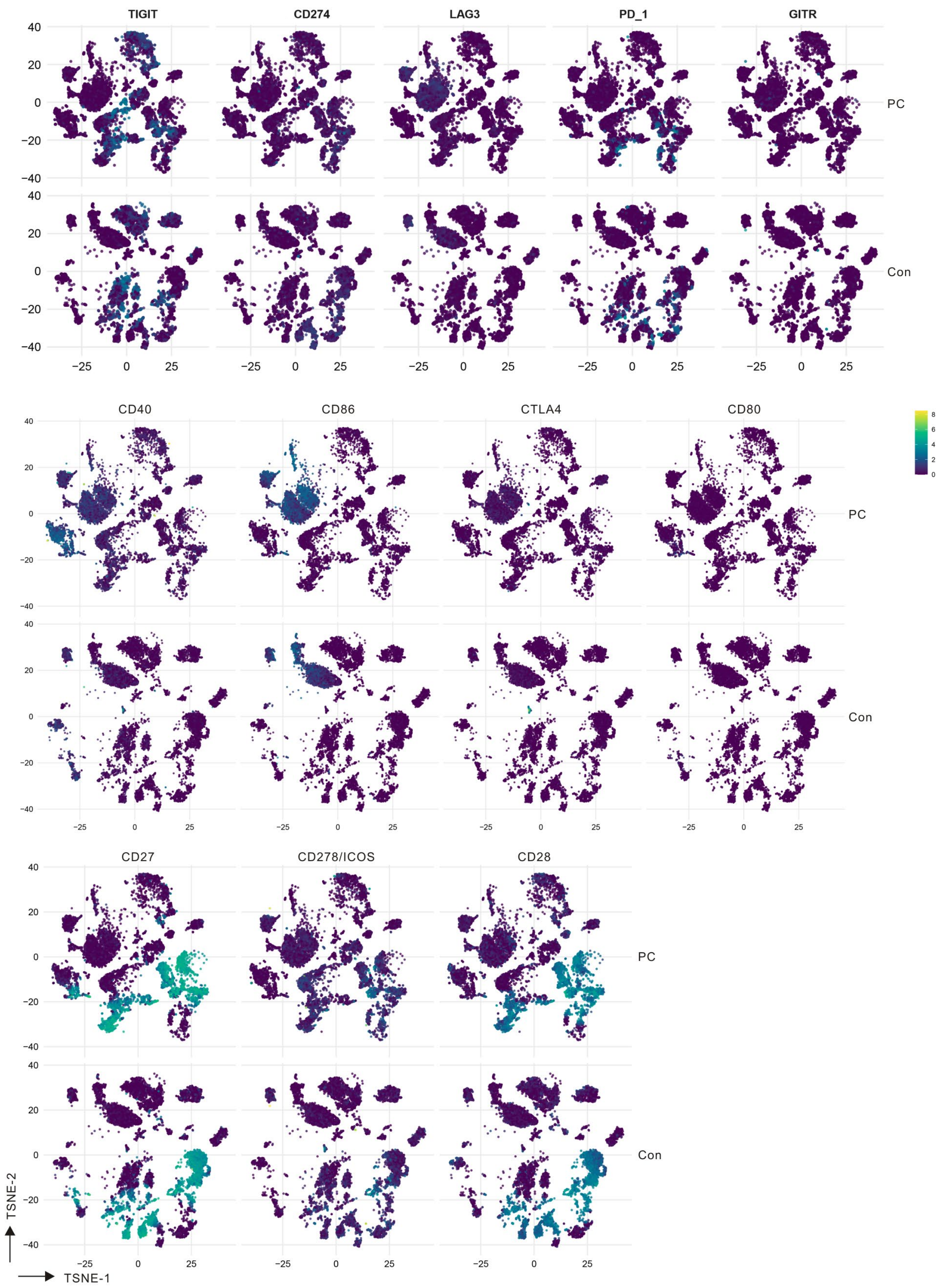

Figure S11 T-SNE plot of mass cytometry showing the selected markers in PC and control group.

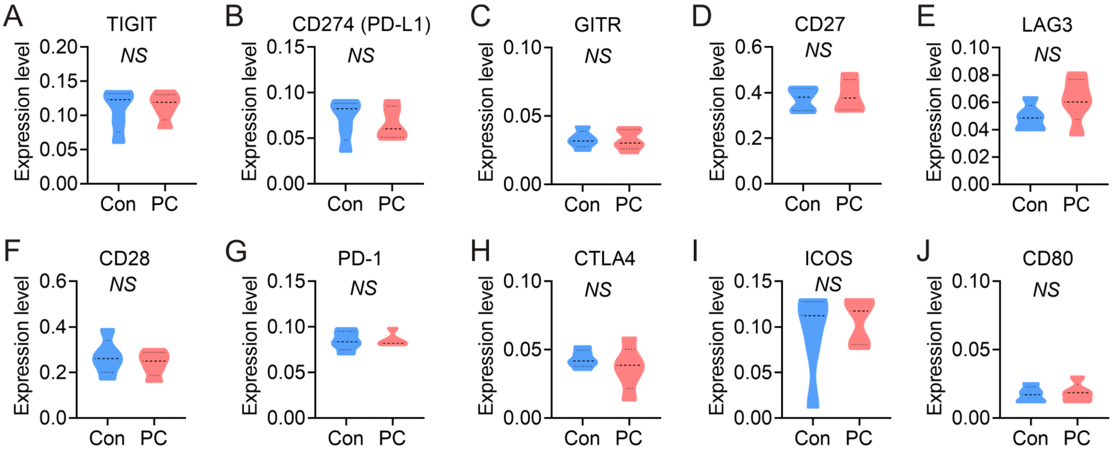

Figure S12 Protein expression level of selected markers in PC and control group. (A) TIGIT. (B) PD-L1. (C) GITR. (D) CD27. (E) LAG3. (F) CD28. (G) PD-1. (H) CTLA4. (I) ICOS. (J) CD80. One-way analysis of variance Wilcoxon rank sum test was used.
