# Supplementary material for: High-dimensional single-cell analysis unveils distinct immune signatures of peripheral blood in patients with pancreatic ductal adenocarcinoma
Source: Front Endocrinol (Lausanne). 2023 Jun 6;14:1181538. doi: 10.3389/fendo.2023.1181538 (PMC10281055; doi:10.3389/fendo.2023.1181538)
Supplement: Supplementary file 2 [file Table_1.doc]

**High-dimensional Single-cell analysis Unveils Distinct** **Immune Signatures of Peripheral Blood in Patients with Pancreatic Ductal Adenocarcinoma**

Yu Pan^1#^, Jianfeng Gao^1#^, Jiajing Lin^1#^, Yuan Ma^1^, Zelin Hou^1^, Yali Lin^1^, Shi Wen^1^, Minggui Pan^2^, Fengchun Lu^1*^, Heguang Huang^1*^

**Supplementary Materials**

**(Table S1 – S8)**

| **Table S1.** Clinical characteristics of patients with PDAC | | | | | | | | | | | | |
| --- | --- | --- | --- | --- | --- | --- | --- | --- | --- | --- | --- | --- |
|  | | **PC1** | | **PC2** | | | **PC3** | | **PC4** | | **PC5** | |
| Gender | | Female | | Female | | | Male | | Male | | Male | |
| Age (years) | | 60 | | 60 | | | 66 | | 67 | | 61 | |
| Tumor site | | Head | | Neck | | | Tail | | Tail | | Head | |
| Procedure | | PD | | DP | | | DP | | LE | | PD | |
| TNM-stage | | T4N2M0 | | T1N0M0 | | | T3N1M0 | | T2N0M1 | | T1N0M0 | |
| Grading | | G2 | | G3 | | | G2 | | G3 | | G2 | |
| Vascular invasion | | Yes | | Yes | | | Yes | | Yes | | No | |
| Chemotherapy | | None | | FOLFIRINOX | | | nab-P+Gem | | nab-P+Gem | | nab-P+Gem | |
| OS (months) | | 5 | | 15 | | | 18 | | 17 | | 21 | |
| State | | Dead | | Dead | | | Dead | | Survival | | Dead | |
|  |  | | **PC6** | | **PC7** | **PC8** | | **PC9** | | **PC10** | |  |
|  | Gender | | Female | | Female | Female | | Male | | Male | |  |
|  | Age (years) | | 64 | | 56 | 67 | | 61 | | 58 | |  |
|  | Tumor site | | Head | | Neck | Head | | Tail | | Head | |  |
|  | Proceduce | | LE | | LE | LE | | DP | | PD | |  |
|  | TNM-stage | | T4NxM1 | | T4NxM1 | T4NxM1 | | T2N0M0 | | T2N1M0 | |  |
|  | Grading | | G3 | | G2 | G3 | | G2 | | G2 | |  |
|  | Vascular invasion | | Yes | | Yes | Yes | | No | | No | |  |
|  | Chemotherapy | | nab-P+Gem | | nab-P+Gem | nab-P+Gem | | None | | nab-P+Gem | |  |
|  | OS (months) | | 12 | | 14 | 31 | | 15 | | 16 | |  |
|  | State | | Survival | | Survival | Dead | | Dead | | Dead | |  |
|  | Abbreviations: PD, pancreaticoduodenectomy; DP, distal pancreatectomy; LE, Laparoscopic exploration; OS, overall survival time; nab-P+Gem, nab-paclitaxel plus gemcitabine | | | | | | | | | | |  |

| **Table S2.** Baseline characteristics of healthy controls | | | | | | | | | |
| --- | --- | --- | --- | --- | --- | --- | --- | --- | --- |
|  | | Con1 | | Con2 | | Con3 | Con4 | Con5 |  |
| Gender | | Male | | Female | | Male | Female | Male |  |
| Age (years) | | 42 | | 23 | | 78 | 77 | 65 |  |
|  |  | |  | | | | | | |
|  | | Con6 | | | Con7 | Con8 | Con9 | Con10 |  |
| Gender | | Female | | | Female | Male | Female | Female |  |
| Age (years) | | 55 | | | 60 | 62 | 50 | 57 |  |

| **Table S3.** Baseline characteristics of PDAC patients and healthy controls | | | | |
| --- | --- | --- | --- | --- |
|  | PDAC patients |  | Healthy controls | p-Value |
|  | (n = 10) |  | (n = 10) |
| Gender |  |  |  |  |
| Male | 5 |  | 4 | 1.000 |
| Female | 5 |  | 6 |  |
| Age (years) |  |  |  |  |
| Median (range) | 62 (56-67) |  | 57 (23-78) | 0.606 |

| **Table S4.** The marker genes and the panel used in mass cytometry (CyTOF). | | | |
| --- | --- | --- | --- |
| **List** | **Label** | **Marker** | **Clone** |
| 1 | Y89 | CD45 | HI30 |
| 2 | 174Yb | CD4 | SK3 |
| 3 | 146Nd | CD8A | RPAT8 |
| 4 | 148Nd | CD14 | RMO52 |
| 5 | 209Bi | CD16 | 3G8 |
| 6 | 162Dy | FOXP3 | PCH101 |
| 7 | 144Nd | CD38 | HIT2 |
| 8 | 160Gd | CD28 | CD28.2 |
| 9 | 143Nd | CD278/ICOS | C398.4A |
| 10 | 176Yb | CD127/IL-7Ra | A019D5 |
| 11 | 154Sm | TIM3 | F382E2 |
| 12 | 161Dy | CD152/CTLA-4 | 14D3 |
| 13 | 173Yb | Granzyme B | GB11 |
| 14 | 164Dy | FAS | DX2 |
| 15 | 151Eu | CD103 | BerACT8 |
| 16 | 142Nd | CD40 | 5C3 |
| 17 | 149Sm | CD25/IL-2R | 2A3 |
| 18 | 150Nd | LAG3 | 11C3C65 |
| 19 | 175Lu | CD274 | 29E.2A3 |
| 20 | 168Er | Ki67 | Ki-67 |
| 21 | 159Tb | GITR | 621 |
| 22 | 158Gd | OX-40 | ACT35 |
| 23 | 167Er | CD27 | O323 |
| 24 | 155Gd | PD-1 | EH12.2H7 |
| 25 | 153Eu | TIGIT | MBSA43 |
| 26 | 166Er | CD24 | ML5 |
| 27 | 145Nd | CD163 | GHI/61 |
| 28 | 170Er | HLA-DR | L243 |
| 29 | 156Gd | CD86 | IT2.2 |
| 30 | 172Yb | CX3CR1 | 2A91 |
| 31 | 163Dy | CD172a/b/SIRPa/b | SE5A5 |
| 32 | 114Cd | CD3 | UCHT1 |
| 33 | 113Cd | CD206/MMR | 15-2 |
| 34 | 116Cd | CD335/NKp46 | 9E2 |
| 35 | 110Cd | CD19 | HIB19 |
| 36 | 141Pr | CD1c | L161 |
| 37 | 152Sm | CD204 | 7C9C20 |
| 38 | 171Yb | CCL22 | 57203 |
| 39 | 112Cd | CD62L/L-selectin | DREG-56 |
| 40 | 111Cd | CD11b | M1/70 |
| 41 | 106Cd | CD11c | Bu15 |
| 42 | 147Sm | SIGLEC15 | 1026113 |
| 43 | 165Ho | TREM2 | 237920 |
| 44 | 169Tm | CD80 | 2D10 |

| **Table S5.** Baseline characteristics of PDAC patients and healthy controls | | | | |
| --- | --- | --- | --- | --- |
|  | PDAC patients |  | Healthy controls | p-Value |
|  | (n = 50) |  | (n = 50) |
| Gender |  |  |  |  |
| Male | 28 |  | 27 | 0.841 |
| Female | 22 |  | 23 |  |
| Age (years) |  |  |  |  |
| Median (range) | 58 (41-74) |  | 54 (35-72) | 0.069 |

| **Table S6.** Clinicopathologic features of PDAC patients and non-PDAC participants in training cohort | | | |
| --- | --- | --- | --- |
|  | PDAC (n = 45) | Pancreatic benign diseases (n = 20) | Healthy controls (n = 30) |
| Sex |  |  |  |
| Male | 24 (53.3%) | 9 (45%) | 18 (60%) |
| Female | 21 (46.7%) | 11 (55%) | 12 (40%) |
| Age (years) |  |  |  |
| Mean | 60.3 | 52.6 | 58.4 |
| Range | 43-80 | 21-76 | 37-78 |
| Hypertension | 13 (28.9%) | 5 (25%) | 3 (10%) |
| Diabetes | 10 (22.2%) | 2 (10%) | 3 (10%) |
| CA19-9 value |  |  |  |
| > 37 U/ml | 36 (80%) | 2 (10%) * | 0 (0.0) * |
| ≤ 37 U/ml | 9 (20%) | 18 (90%) | 30 (100%) |
| Tumor location |  |  |  |
| Pancreatic head | 29 (64.4%) | NA | NA |
| Other locations | 16 (35.6%) | NA | NA |
| Procedure |  |  |  |
| PD | 23 (51.1%) | NA | NA |
| DP | 15 (33.3%) | NA | NA |
| TP | 4 (8.9%) | NA | NA |
| Without operation | 3 (6.7%) | NA | NA |
| TNM-stage |  |  |  |
| Stage I | 6 (13.3%) | NA | NA |
| Stage II | 16 (35.6%) | NA | NA |
| Stage III | 20 (44.4%) | NA | NA |
| Stage IV | 3 (6.7%) | NA | NA |
| Resection margin | |  |  |
| R0 | 20 (44.4%) | NA | NA |
| R1 | 22 (48.9%) | NA | NA |
| Without operation | 3 (6.7%) | NA | NA |
| Histologic grade |  |  |  |
| G1 | 3 (6.7%) | NA | NA |
| G2 | 30 (66.6%) | NA | NA |
| G3 | 9 (20%) | NA | NA |
| Without operation | 3 (6.7%) | NA | NA |
| Vascular invasion | |  |  |
| Yes | 17 (37.8%) | NA | NA |
| No | 28 (62.2%) | NA | NA |
| Chemotherapy |  |  |  |
| Yes | 21 (46.7%) | NA | NA |
| No | 24 (53.3%) | NA | NA |
| Abbreviations: PD, pancreaticoduodenectomy; DP, distal pancreatectomy; TP, total pancreatectomy; NA, not available. *, P < 0.01 (compared to PDAC group). | | | |

| **Table S7.** Values of 16 serum immune proteins in patients with PDAC and in non-PDAC subjects | | | |
| --- | --- | --- | --- |
|  | PDAC (n=45) |  | non-PDAC (n=50) |
|  | Median (pg/ml) |  | Median (pg/ml) |
| sBTLA | 66 (31-339.9) |  | 75.26 (31-441) |
| sCD27 | 2093 (294.44-15036) |  | 1511.5 (452.38-4831) |
| sCD28 | 1180 (357.75-22798) |  | 1194.5 (306.44-15066) |
| sTIM-3 | 4585 (1332-19913) |  | 2026.5 (806.2-3492) ** |
| sHVEM | 3098 (807.14-6977) |  | 2425.5 (478.74-4475) |
| sCD40 | 525.89 (246.28-2174) |  | 443.29 (238.69-1057) |
| sGITR | 11.21 (3-369.22) |  | 11.14 (3-757.41) |
| sLAG-3 | 134847 (27481-357881) |  | 112625 (25610-331911) |
| sTLR-2 | 763.08 (190.18-1987) |  | 493.35 (179.24-1901) |
| sGITRL | 62.69 (22-684.51) |  | 33.05 (22-1405) |
| sPD-1 | 347.18 (171.74-1077) |  | 273.1 (127.28-851.39) |
| sCTLA-4 | 16.13 (5-164.15) |  | 15.82 (5-178.97) |
| sCD80/B7-1 | 18.22 (12-124.7) |  | 18.1 (12-149.99) |
| sCD86/B7-2 | 279.01 (100-1542) |  | 246.39 (116.28-1753) |
| sPD-L1 | 21.97 (7.92-131.66) |  | 20.135 (11.59-154.32) |
| sICOS | 174.32 (60-2787) |  | 152.085 (60-1881) |

**, P < 0.001 (compared to PDAC group).

| **Table S8.** Clinicopathologic features of PDAC patients and non-PDAC participants in validation cohort | | | |
| --- | --- | --- | --- |
|  | PDAC (n = 53) | Pancreatic benign diseases (n = 22) | Healthy controls (n = 25) |
| Sex |  |  |  |
| Male | 34 (64.1%) | 9 (40.9%) | 11 (44%) |
| Female | 20 (35.9%) | 13 (59.1%) | 14 (56%) |
| Age (years) |  |  |  |
| Mean | 61.1 | 54.3 | 50.4 |
| Range | 35-76 | 26-75 | 22-74 |
| Hypertension | 18 (33.9%) | 6 (27.3%) | 3 (12.0%) |
| Diabetes | 10 (18.8%) | 2 (9.1%) | 1 (4.0%) |
| CA19-9 value |  |  |  |
| > 37 U/ml | 44 (83%) | 2 (9.1%) * | 0 (0.0%) * |
| ≤ 37 U/ml | 9 (17%) | 20 (90.9%) | 25 (100%) |
| Tumor location |  |  |  |
| Pancreatic head | 34 (64.2%) | NA | NA |
| Other locations | 19 (35.8%) | NA | NA |
| Procedure |  |  |  |
| PD | 26 (49.1%) | NA | NA |
| DP | 16 (30.2%) | NA | NA |
| TP | 5 (9.4%) | NA | NA |
| Without operation | 6 (11.3%) | NA | NA |
| TNM-stage |  |  |  |
| Stage I | 8 (15.1%) | NA | NA |
| Stage II | 26 (49.1%) | NA | NA |
| Stage III | 15 (28.3%) | NA | NA |
| Stage IV | 4 (7.5%) | NA | NA |
| Resection margin | |  |  |
| R0 | 19 (35.8%) | NA | NA |
| R1 | 28 (52.8%) | NA | NA |
| Without operation | 6 (11.3%) | NA | NA |
| Histologic grade |  |  |  |
| G1 | 2 (3.8%) | NA | NA |
| G2 | 42 (79.2%) | NA | NA |
| G3 | 9 (17.0%) | NA | NA |
| Without operation |  | NA | NA |
| Vascular invasion | |  |  |
| Yes | 20 (37.7%) | NA | NA |
| No | 33 (62.3%) | NA | NA |
| Chemotherapy |  |  |  |
| Yes | 25 (47.2%) | NA | NA |
| No | 28 (52.8%) | NA | NA |
| Abbreviations: PD, pancreaticoduodenectomy; DP, distal pancreatectomy; TP, total pancreatectomy; NA, not available. *, P < 0.01 (compared to PDAC group). | | | |
